# Forward Precision Medicine: Micelles for Active Targeting Driven by Peptides

**DOI:** 10.3390/molecules26134049

**Published:** 2021-07-02

**Authors:** Filippo Prencipe, Carlo Diaferia, Filomena Rossi, Luisa Ronga, Diego Tesauro

**Affiliations:** 1 Institute of Crystallography (IC) CNR, Via Amendola 122/o, 70126 Bari, Italy; filippo.prencipe@ic.cnr.it; 2 Department of Pharmacy and Interuniversity Research Centre on Bioactive Peptides (CIRPeB), University of Naples “Federico II”, via Mezzocannone 16, 80134 Naples, Italy; carlo.diaferia@unina.it (C.D.); filrossi@unina.it (F.R.); 3 Institut des Sciences Analytiques et de Physico-Chimie Pour l’Environnement et les Matériaux, Université de Pau et des Pays de l’Adour, E2S UPPA, CNRS, IPREM, 64053 Pau, France; luisa.ronga@univ-pau.fr

**Keywords:** micelles, peptide targeting, drug delivery, precision medicine, nanomedicine

## Abstract

Precision medicine is based on innovative administration methods of active principles. Drug delivery on tissue of interest allows improving the therapeutic index and reducing the side effects. Active targeting by means of drug-encapsulated micelles decorated with targeting bioactive moieties represents a new frontier. Between the bioactive moieties, peptides, for their versatility, easy synthesis and immunogenicity, can be selected to direct a drug toward a considerable number of molecular targets overexpressed on both cancer vasculature and cancer cells. Moreover, short peptide sequences can facilitate cellular intake. This review focuses on micelles achieved by self-assembling or mixing peptide-grafted surfactants or peptide-decorated amphiphilic copolymers. Nanovectors loaded with hydrophobic or hydrophilic cytotoxic drugs or with gene silence sequences and externally functionalized with natural or synthetic peptides are described based on their formulation and in vitro and in vivo behaviors.

## 1. Introduction

The controlled and specific release of chemical entities plays a key role in different technology applications, often avoiding harmful high concentrations and allowing chemicals to exist for a prolonged period of time. Especially in pharmaceutical sciences, the controlled release of active pharmaceutical ingredients (APIs) is a central topic. In drug administration, two main problems are observed: drug degradation and the lack of API selectivity. In addition, the second disadvantage is the several side effects in healthy tissue, which decrease bioavailability and the percentage of drug accumulated in the pathological tissue. The use of specific carriers can prevent both inconveniences. In recent decades, drugs have been loaded in microcapsules [1], lipoproteins [2], non-ionic surfactant vesicles (niosomes) [3], lipid particles [4], liposomes [5], micelles [6], betacyclodextrin [7] carriers and, more recently, carbon nanotubes. Additionally, different loading strategies were explored, including cavity loading, matrix loading, molecular-level loading and surface loading.

Nanoparticles can be engineered tuning their size, shape, chemical and physical properties, etc., in order to program them depending on loading, stimuli responsiveness and release and targeting requirements. Delivering the drug precisely and safely to its target cells is the most important goal of novel drug delivery systems. Moreover, controlled release and achievement of the maximum therapeutic result remain the main targets in drug carriers. In chemotherapy, the employment of delivery systems is crucial because of the severe side effects of the drugs used to fight tumor cells, due to nonspecific targeting by anticancer agents. The main challenge is to discriminate among the cancerous cells and the normal body cells. The delivery system has to be designed in such a way in order to identify the cancer cells and consequentially inhibit their growth and proliferation. Between the different classes of systems proposed for this purpose, we present, here, an overview of surfactant and polymer micelles as well as different peptides used for the decoration of micelles enabling active targeting of a system. We focused our attention on several copolymers which are able to form aggregates enabling them to carry drugs, highlighting the enhanced properties of specific targeting and cell penetration.

### 1.1. Micelle-Like Carriers

Micelles are colloidal dispersions consisting of discrete particles (disperse phase) having sizes within the range of 5 to 100 nm distributed in a continuous phase (dispersion medium).

**Figure 1:**
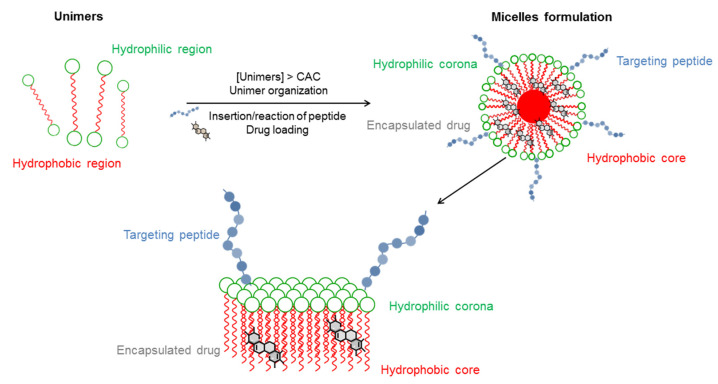
Graphical and general representation of unimer organization in peptide-targeting drug-loaded micelle formulations.

When amphiphilic compounds are dissolved in water under a determinate temperature and concentration, they start to self-orientate the hydrophilic moiety at the surface so that the hydrophobic regions are removed from the aqueous environment. These physicochemical phenomena lead to the formation of aggregates. The aggregation process is driven by the water exclusion effect, hydrophobic and van der Waals attractions between hydrocarbon moieties and, in the opposite direction, by electrostatic and steric hydrophilic moiety repulsions. The equilibrium between these intermolecular forces controls the micellar shape (cylindrical, rod-like and spherical shape). Micelles are formulated by amphiphilic unimers able to aggregate up to a minimum concentration. At low concentration, they exist as unimers, whereas aggregation takes place only when the concentration increases (see Figure 1). The lowest concentration at which aggregates appear is called the critical micellar concentration (CMC). This value is crucial for micellar employment in medical applications. Micelles may be engineered tuning the aggregation condition. Therefore, many factors influence micellar stability, inducing stimulus-sensitive aggregation in order to respond to local stimuli characteristics of the pathological site.

From a structural point of view, micelles are formed by two regions: the hydrophobic core and the hydrophilic shell or corona (Figure 1). This organization elucidates the use of micelles in drug delivery. Indeed, the core can incorporate lipophilic molecules which are solubilized in the bulk solvent. The favorable interactions with the solvent of the head groups forming the shell allow supramolecular dissolution of the API, improving bioavailability and pharmacokinetics profiles. On the contrary, water-soluble drugs may be absorbed on a hydrophilic shell via electrostatic interactions and semi-polar molecules lying along the amphiphilic molecule in an intermediate position.

### 1.2. Micelle Composition

According to their pharmaceutical application, micelle formulations have to be designed to save their integrity for a specific length of time in circulating blood to deliver their payloads selectively on the pathological sites. They should hold down the aggregation at the lowest possible unimer concentration (low CMC), but they should be able to release drugs at the right moment in the desired tissues. One method to improve blood stability is to reduce the rate of nonspecific recognition. In particular, the hydrophobic segments of the micellar core control drug release, whereas the hydrophilic moieties provide steric stability and avoid rapid uptake by the immune system. Indeed, the systemic circulation time was found to be affected by the surface of delivery carriers. Charged systems with hydrophobic surfaces have more tendency to opsonization and are consequently taken up by the reticuloendothelial system (RES). Another factor to take into account in micelle design is the degradation of the carrier in response to pH and/or temperature. Many pathological processes in various tissues and organs are accompanied by a local temperature increase (by 2–5 °C) and/or a pH decrease by pH 1–2.5 units (acidosis). The pH gradient is predicted by hypoxia that upregulates glycolysis, followed by the formation of lactate and protons in the extracellular microenvironment. In particular, the tumor extracellular environment is more acidic (pH 6.2–6.9) than normal tissues (pH 7.4) [8], while the pH value in cellular endo/lysosomes is even lower (4.0–6.0) [9]. Even if these variations in in vivo environments represent an issue for the formulation and optimization of the systems, these properties can be exploited increasing the selective release. pH-sensitive micelles can be developed by two main strategies. One approach is the exploitation of pH-labile chemical bond conjugating drugs, through hydrazone, *cis*-acotinyl and acetal bonds. The presence of acid-labile linkages allows releasing a drug in relatively acidic extracellular fluids or in endosomes or lysosomes in tumor cells. The second approach is through attached groups in copolymers, such as amines or carboxylic acids.

The presence of amine groups in the core being uncharged at high pH values makes the polymer hydrophobic, which becomes hydrophilic upon protonation at low pH values. This variation induces physical dissociation or interior structural change and drug release. Many examples of basic groups are reported such poly(histidine) or poly(Lysine). At the same time, acidic groups such as carboxylic acids can induce variation such as poly d,l-lactic acid protonated at low pH.

Another parameter that improves drug release, inducing the disaggregation of nanoparticles in reductive environments, is the presence of a disulfide bond in amphiphilic molecules. This bond is stable in the extracellular environment, but it can be cleaved rapidly by glutathione in the cytoplasm and nucleus.

## 2. Passive and Active Targeting

Micelles reach the target producing an accumulation of the drug via the enhanced permeability and retention (EPR) effect based on blood vessel overgrowth in tumor tissues [10].

Aggregates in the range of 10–500 nm remain in the circulation for an extended period through the compromised leaky vasculature, which is specific for solid tumors, infarcts, infections and inflammations [11]. This property allows the accumulation of drug-based nanomaterials in tumor cells and their retention for a long period of time (days to weeks).

However, the EPR effect is highly heterogeneous in the clinic experience depending on cancer typology, patients affected by the same tumors and even within the same tumor mass [12]. Moreover, chemotherapeutics accumulation in tumor tissue via stabilized nanoparticles seems to be a key factor but does not assure an improvement in the therapeutic effect. There is evidence that liposomes loaded with doxorubicin (DOX) are able to produce a higher drug concentration in the tumor vasculature but with no appreciable increase in the intracellular concentration of the drug, thus not producing an improvement in the therapeutic effect compared to the free drug. Consequently, active targeting can improve the internalization process in tumor cells [13]. This approach takes advantage of the specific interaction of bioactive molecules capable of selectively identifying receptors overexpressed in tumor cells and with reduced expression in nearby normal cells. Ligand -labeled nanocarriers are designed based on the affinity between the ligands and receptors expressed on pathological cell membranes. Targeting ligands influence nanocarriers’ circulation time, cellular uptake, affinity and extravasation. These molecules include monoclonal antibodies, nucleic acids (deoxyribonucleic acid/ribonucleic acid (DNA/RNA) aptamers), peptides or proteins and even vitamins or carbohydrates. Although monoclonal antibodies or antibody fragments provide a large number of advantages such as a high degree of specificity, elevated binding affinities and their own therapeutic effects, many drawbacks can be listed such as limited stability and difficulty of storage. Moreover, immunogenicity problems can arise after one administration, and extravasation can occur due to their large molecular weight. Another disadvantage is the high cost of these targeting systems.

Several non-antibody ligands can graft nanoparticles to deliver them to cancer tissues. Among these ligands, peptides can play a relevant role, being a very versatile tool. They can be designed to bind tumor-related targets because of their infinite sequence and structure potentials. Their synthesis is less expensive than antibody production, and it is expected to yield fewer problems linked to immunogenicity. Peptides can promote membrane penetration with the grafted aggregates [14] or recognize overexpressed receptors on the cell membrane [15].

The most relevant drawback of these systems is the metabolic degradation of peptides that can be extremely sensitive to protease actions. This issue can be overcome to improve metabolic stability and pharmacokinetics by designing peptide sequences with amino acids with d configurations and/or uncoded amino acids or by cyclization. 

In most cases, the bioactive peptide may be coupled during nanostructure preparation to an amphiphilic moiety according to a pre-functionalization strategy. Alternatively, peptide surface binding can be performed after nanostructures have been obtained according to a post-functionalization approach.

**Scheme 1:**
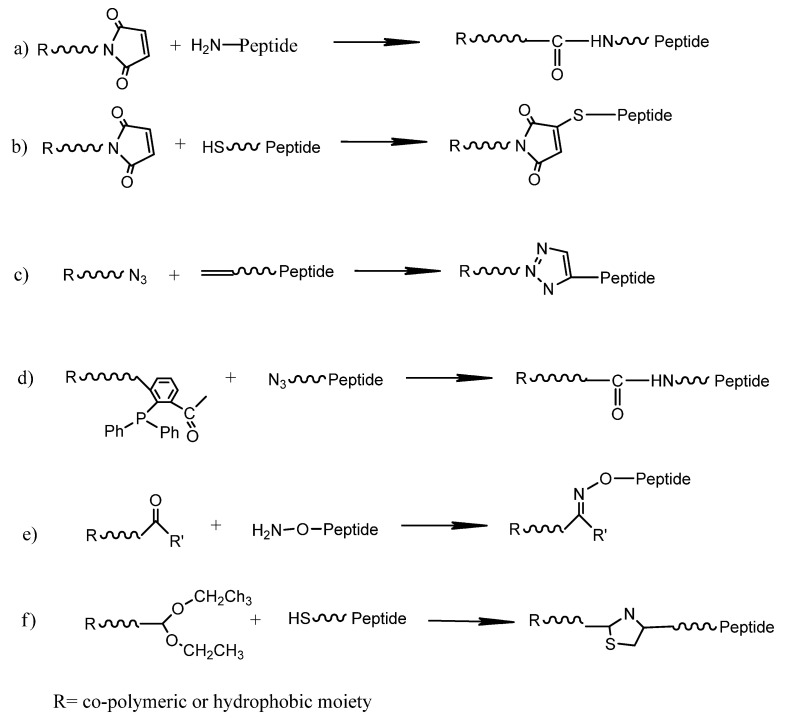
Chemical reactions for peptide coupling: (**a**) amine-*N*-hydroxysuccinamide coupling method, (**b**) Michael addition, (**c**) Cu(I)-catalyzed Huisgen cycloaddition (CuAAC) (click chemistry), (**d**) Staudinger ligation, (**e**) oxime bond arrangement and (**f**) thiazolidine ring formation.

This strategy is pursued by functionalizing the *N*-terminus of peptides with different organic groups able to bind the micelle surface in mild conditions. The principal pathways are based on the following: (a) the amine-*N*-hydroxysuccinamide coupling method, (b) Michael addition, (c) Cu(I)-catalyzed Huisgen cycloaddition (CuAAC) (click chemistry), (d) Staudinger ligation, (e) oxime bond arrangement, (f) thiazolidine ring formation. Each coupling reaction is reported in Scheme 1.

### 2.1. Cell-Penetrating Peptides (CPPs)

CCPs are a class of peptides able to penetrate the cellular membrane [16]. They are usually constituted by up to 30 amino acids and, in all sequences, predominate residues bearing a positive charge. CCPs can be rationally designed or derived from protein transduction domains. They have established themselves as delivery vehicles due to their capability of autonomous and receptor-independent intracellular translocation, having potential applications in disease diagnosis and therapy, including cancer, inflammation, diabetes, central nervous system disorders and ocular disorders [17,18].

Many CPPs were designed from sequences of membrane-interacting proteins, such as fusion proteins, signal peptides, transmembrane domains and antimicrobial peptides. Short sequences (called protein transduction domains or PTDs) were selected among these, such as the third helix of the homeodomain of antennapedia called penetratin [19]. The Tat peptide (48–60) was derived from the trans activating transcription protein (Tat) of human immunodeficiency virus-type 1 (HIV-1) [20]. It penetrates the cell because it has a specific motif or helical structure [21].

Synthetic CPPs are based on the role of positive charges in the translocation process. Investigations performed on Tat and penetratin revealed that the role of positive charges is crucial for translocation. Among the cationic polypeptides, polyarginines (Poly-R) display a higher efficiency compared with polyhistidines or polylysines [22]. The chimeric CPPs group is generally renowned as a transition from natural to synthetic CPPs since they contain sequences from two or more different naturally occurring proteins. Examples include amphipathic peptide (CADY) (20 amino acids) which combines an aromatic residue (tryptophan) and a cationic residue (arginine) [23]. Depending on their secondary structures, CPPs can be classified based on conformation. Actually, they are divided into linear and cyclic CPPs. Up to date, studies have confirmed that cyclic CPPs show improvements if compared to their linear matching parts. Definitely, the comparison between linear and cyclic CPPs highlights that the latter have higher cell permeability and higher affinity with the target receptor on the cell. Consequently, the transmembrane ability is further increased through receptor-mediated uptake [24]. Bechara et al. and Qian et al. [25,26] explained that linear CPPs are more sensitive to proteolytic hydrolysis than cyclic CCPs, resulting in poor pharmacokinetic properties in vivo.

Based on differences in physicochemical properties, CPPs can be classified into three subgroups, specifically: cationic, amphipathic and hydrophobic. Below physiological pH conditions, the positive charge of cationic CPPs confirms the excellent affinity with the cytoplasmic membrane. They interact with the negatively charged cell membrane glycoproteins through electrostatic interaction and then are internalized into the cell through an independent receptor mechanism. Most cationic CPPs typically contain more than five positively charged amino acids such as poly-arginine responsible for the highest cellular uptake aptitude and therapeutic potential, as shown by Borrelli et al. [27]. Chu et al. also revealed that the internalization capacity of oligoarginine increases with its length (from R8 to R10) [28]. Higher values will have irreversible side effects on the cells and reduce the overall delivery efficiency, as reported by Verdurmen and Brock [29].

### 2.2. Receptor-Recognizing Peptides

Receptors can mediate the process of endocytosis, facilitating intracellular delivery of the projected payload. The most popular class of peptides is able to target nanoparticles to integrin receptors.

**Figure 2:**
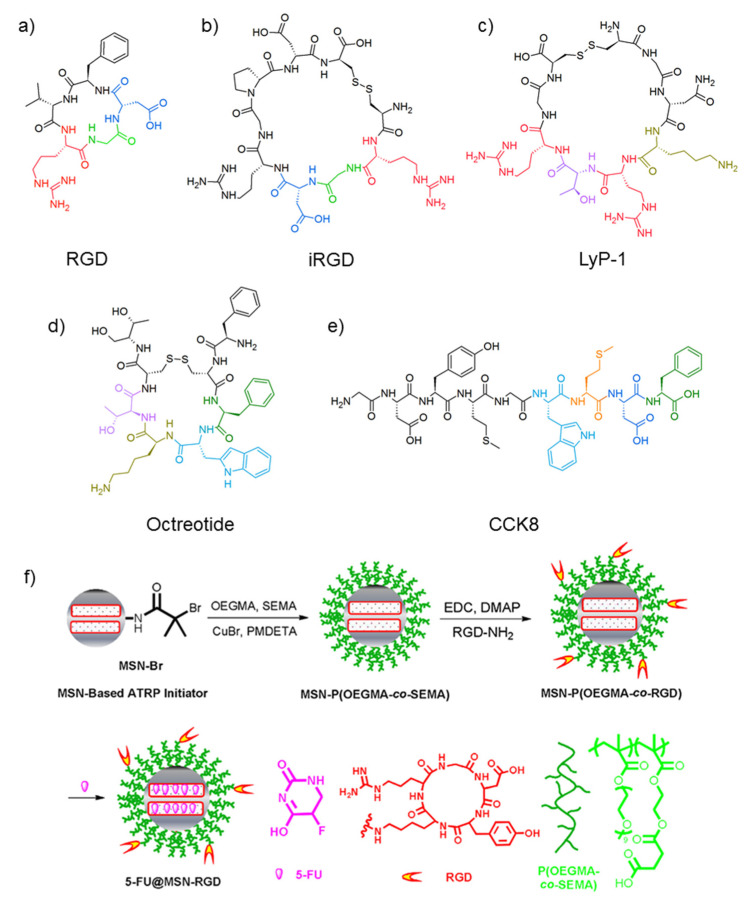
Chemical structure of peptide receptor sequences (**a**–**e**). Recognition residues are reported in colors; (**f**) synthetic routes employed for obtaining mesoporous silica nanoparticles and their analogues loaded with 5-FU (5-FU@MSN-RGD). (Reprinted with permission from [39]).

Integrins are heterodimeric transmembrane protein families, and among these integrins, ανβ3 and ανβ5 are overexpressed in endothelial cells of many types of cancers and are emerging as markers of tumoral neo-angiogenesis. Indeed, many investigations are reported in the field of ανβ3 integrin-mediated bioactive RGD motif (Figure 2a)-containing peptides [30].

The prototype of RGD motif-containing peptides is cilengitide, the first anti-angiogenic small molecule, specifically a cyclic pentapeptide, developed by Kessler, with high activity and selectivity towards ανβ3 integrin receptors [31]. Notably, the constraint deriving from the cyclic structure of this peptide induces an improvement in binding properties, conferring rigidity and selectivity to the structure. The first aggregate grafted with RGD-containing peptides was formulated to deliver DOX to the vasculature of colon cancer, improving its efficacy [32,33]. Since 2003, many patents and more than 500 articles have been published on RGD-labeled nanoparticles and nanoaggregates delivering hydrophilic drugs such as DOX [34], but also with inorganic chemotherapeutics such as *cis*platin (CDDP) [35] or hydrophobic drugs such as paclitaxel (PXL) [36,37]. Moreover, nanoparticles were prepared to co-deliver two therapeutics such as siRNA and docetaxel (DTX) [38] or combretastatin A4 (CA4) and DOX. The preparation scheme is shown in Figure 2f [39]. In addition, RGD sequences are able to decorate mesoporous silica nanoparticles, improving the efficacy of 5-Fluorouracil [40], and join to folate, allowing targeting paclitaxel (PTX) to human breast cancer cells (MCF-7) [41].

Based on cilengitide, other homologue sequences (Val residue replaced by Lys) were designed to selectively deliver diagnostics and therapeutics to cancer cells [42]. Ruoslahti’s group selected another sequence from a library displayed on T7 phage 10^9^ to recognize tumor blood vessels in experimental metastasis mouse models of human prostate cancer [43]. The nine-amino acid cyclic peptide CRGDRGPDC (iRGD, Figure 2b) includes a vascular homing motif RGD, is able to target tumor cells and is a motif CendR ((R/K)XX(R/K) peptide that induces extravasation and tissue penetration via a mechanism that involves cell internalization. The peptide loses its affinity for the primary tumor integrin receptor after proteolytic cleavage and acquires affinity for Neuropilin1 receptor due to the CendR motif [44]. In a previous study, the same group identified, by phage display [45], cyclic tumor-homing peptide, LyP-1 (sequence: CGNKRTRGC, Figure 2c) containing a tumor-homing motif and a cryptic CendR motif (KRTR) able to be internalized by Neuropilin receptors (NRP), modular transmembrane proteins for various forms and isoforms of VEGF and members of the class 3 semaphorin family. NRP1 and NRP 2 are overexpressed on the surface of endothelial cells of angiogenic blood vessels and glioma cells. Later, they also synthesized a truncated form, tLyP-1 (CGNKRTR), which displays an enhanced penetration ability within tumor tissue compared with full-length LyP-1, even when tethered on nanoparticles due to CendR being the free *C*-terminus [46]. Another class of peptides was studied as homing peptides for the recognition of G protein-coupled receptors (GPCRs). GPCRs constitute a membrane protein family involved in the recognition and transduction of signals and small molecule signaling, including peptides, nucleotides and proteins. General structural features show the presence of seven transmembrane helices connected by three intracellular and three extracellular loops. The *N*-terminal domain points toward the extracellular space, and the *C*-terminal is directed to the intracellular space. The ligand binding site for peptides has been identified in the *N*-terminal extra-domain or in the portion of the extracellular loops adjacent to the extracellular moiety of the transmembrane helices. These receptors are overexpressed in human tumors of different origin: somatostatin receptors in neuroendocrine tumors, cholecystokinin (CCK) receptors in medullary thyroid cancer, bombesin receptors in prostate and breast carcinoma, and several other tumors. For all receptors, short peptides deriving from truncated natural sequences have been selected. The endogenous somatostatin that binds five receptors (SSTR1–SSTR5) has a very short biological half-life [47]. Therefore, many somatostatin derivatives have been developed; the most successful analogue is octreotide (OCT, Figure 2d) by Sandoz (now Novartis) [48,49]. OCT is able to induce endocytosis by binding to SSTR2 with high affinity which is not affected when its *N*-terminus is chemically functionalized. This evidence allows employing this sequence as a vector of chelating agents for diagnosis and therapy [50,51], of nanoparticles [52] or of copolymers bearing DOX [53].

CCK2-R is targeted with high affinity by a wide number of CCKs, e.g., CCK8 peptide (Figure 2e), and gastrin derivatives characterized over recent years for the purpose of in vivo receptor targeting for imaging and therapy [54]. NMR studies [55] and theoretical calculations [56] established that receptor binding is promoted by the interaction of the *C*-terminal fragment of the peptide ligand with the receptor *N*-terminal extra-domain, where the peptide *N*-terminus constitutes an anchor point for nanoparticles.

## 3. Peptide Amphiphilic Micelles (PAs)

**Figure 3:**
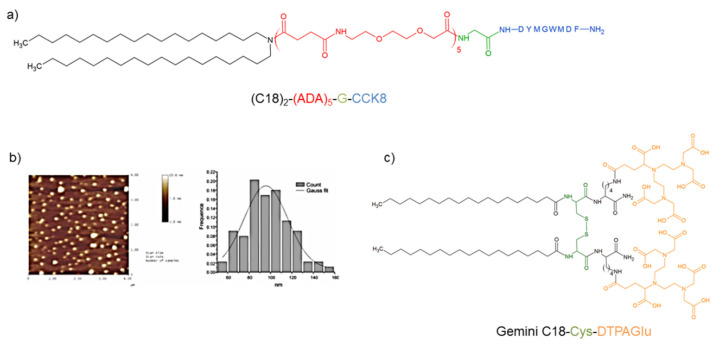
Chemical representation of: (**a**) (C18)_2_(ADA)_5_-G-CCK8, (**b**) AFM microphotos of peptide amphiphile-based aggregates (1 × 10^−5^ mol/kg) and relative diameter histogram distribution (reprinted with permission from [58]), (**c**) gemini C18-Cys-DTPAGlu.

Peptide-grafted surfactant micelles are achieved by the self-assembly of peptide-containing amphiphilic molecules. PA micelles are easy to formulate, and the size and charge of the micelles can be controlled by modulating the hydrophilic and lipophilic balance. These parameters are crucial for their in vivo stability as well as for the circulation half-life. Recently, peptide amphiphilic properties for assembly in nanostructures were also predicted by a combined computational design approach [57]. The hydrophobic moieties are long fatty acids having the length of carbon chains, which is crucial to increase the stability of the aggregates. During self-assembly, hydrophobic segments assemble to form the inner core of the nanostructure, and peptide-containing hydrophilic segments are displaced into the outer surface. In order to increase peptide exposition to promote receptor binding, hydrophilic residues are generally added. For example, oligo ethylene glycol linkers such as 8-amino-3,6-dioxaoctanoic acid (ADA) can fulfill this role, increasing the hydrophilicity without introducing a negative or positive charge. Accardo et al. [58] obtained stable aggregates prompted by the self-assembly of the amphiphile peptide (C18)_2_(ADA)_5_G-CCK8 (Figure 3a) at physiological pH. The resulting micelles presented a CMC value of 2 × 10^−6^ mol·kg^−1^ and a size of >100 nm, as confirmed by AFM characterization (Figure 3b). This aggregate model is able to entrap DOX in an aqueous sphere. Adding this amphiphile peptide to an amphiphilic chelating agent, (C_18_H_37_)_2_NCO(CH_2_)_2_COLys (DTPAGlu)CONH_2_ (DTPAGlu is *N*,*N*-bis[2-[bis(carboxyethyl)amino]ethyl]-l-glutamic acid) in a several molar ratio, mixed micelles were achieved at low ionic strength [59]. The DTPAGlu chelating agent coordinates Gd^3+^ to deliver this ion as a contrast agent for MRI imaging with a high value of relaxivity (18.6 Mm^−1^s^−1^) for the ion. Later, the same group studied the CCK8 conformation on micelle surfaces by circular dichroism (CD) [60]. Supramolecular aggregates obtained by self-aggregation of (C18)_2_(ADA)_5_CCK8, in which the CCK8 peptide is present in a β-sheet conformation, probably due to the inter-peptide hydrogen bonds between amino acid side chains, do not bind in vitro receptor-overexpressing cells. The introduction of three positively charged lysine residues on the *N*-terminal moiety of the peptide induces an electrostatic repulsion between peptides, causing a CCK8 unfolding process. Chemical modifications on the CCK8 *N*-terminus play an important role in stabilizing the peptide active conformation. Indeed, these aggregates possess the ability to bind CCK2-R receptors overexpressed by transfected A431 cells, as indicated by cytofluorimetric studies. The insertion of a Cys residue on the *N*-terminal of CCK8 allows coupling two amphiphilic molecules through a sulfide bridge, obtaining a gemini unimer [C_18_CysL_5_CCK8]_2_ [61]. Adding this molecule to [C18CysDTPAGlu]_2_ (Figure 3c) in a 70:30 molar ratio, the two gemini surfactants aggregate in water solution, forming mixed micelles, with a CMC of 5 × 10^−5^ mol·kg^−1^. The structural characterization performed by small-angle neutron scattering (SANS) demonstrated that the shape and size of micelles are influenced by the temperature: an increase in the temperature leads to a progressive reduction in the size and to a transition from cylindrical structures to ellipsoidal micelles at 50–80 °C. Furthermore, the surface-exposed CCK8 peptide changes its conformation above a transition temperature of approximately 45 °C, switching from a β-sheet to a random coil structure, as indicated by CD measurements. The higher rigidity of the structure with respect to the previously described aggregates brought an increase in the relaxivity value of the gadolinium complex up to 21.5 mM^−1^s^−1^. In order to formulate nanoaggregates to deliver contrast agents with enhanced performance in terms of relaxivity per gadolinium ion, the same group also designed and synthesized octreotide amphiphilic molecules to target SSTR2 [62]. The unimers (OCA-DTPAGlu and OCA-DOTA) contained, in the same molecule, three different functions: a chelating agent (DTPAGlu or DOTA), the octreotide bioactive peptide and a hydrophobic moiety with two 18-carbon atom alkyl chains. Both unimers aggregate in water solution, providing stable micelles at a very low concentration (CMC values of 2.3 × 10^−6^ mol·kg^−1^ and 2.5 × 10^−6^ mol·kg^−1^ for OCA-DTPAGlu and OCA-DOTA, respectively). Fluorescence studies and CD experiments indicate, for both compounds, the complete exposure of octreotide on the micelle surface, and the predominant presence of an antiparallel β-sheet peptide conformation characterized by a β-like turn. The relaxivity value (r_1p_ = 13.9 mM^−1^s^−1^ at 20 MHz and 25 °C), measured for micelles, was obtained by the gadolinium complex OCA-DTPAGlu(Gd). With the same aim, mixed micelles were prepared adding the octreotide peptide surfactant (10% *mol*/*mol*) to amphiphilic molecules containing Gd chelating group (C18)_2_DTPAGlu, (C18)_2_DTPA or (C18)_2_DOTA [63]. Structural investigations demonstrated that, according to a decrease in negative charges in the surfactant head group, a total or a partial micelle-to-vesicle transition is observed by moving from (C18)_2_DTPAGlu to (C18)_2_DOTA. The proton relaxivity of mixed aggregates based on (C18)_2_DTPAGlu(Gd), (C18)_2_DTPA(Gd) and (C18)_2_DOTA(Gd) resulted in being 17.6, 15.2 and 10.0 mM^−1^s^−1^, higher than that measured previously in self-assembled amphiphilic molecules.

The same surfactant design plan was used to synthesize unimers bearing an RGD sequence as a bioactive molecule [64]. The self-assembled aggregates of (C18-ADA_5_-RGD) were loaded with paclitaxel. The resulting aggregates were tested in vitro on A2058 melanoma cells, and the IC_50_ decreased by about 50% in ανβ3 integrin-overexpressing cells where they showed a 4-fold increase in normal cells when compared to free PTX. In order to compare biological results, later on, the same group replaced the RGD sequence with its cyclic analogue cRGDfK [65]. cRGDfK micelles displayed a similar CMC, shape and size. However, cyclic RGD micelles exhibited better targeting efficacy but were less effective with respect to linear RGD micelles as drug delivery systems due to the lower drug solubilization capacity and lesser kinetic stability.

Liang et al. linked an RGD sequence to lauric acid to encapsulate curcumin (CUR), a poorly soluble drug [66]. The lipopeptide is able to self-assemble in spherical micelles with a diameter of 30 nm, increasing CUR solubilization by three orders of magnitude. CUR-loaded LP micelles exhibited much higher cell proliferation inhibition than free CUR on human cervix carcinoma cells (HeLa) (IC_50_ values of 22.5 vs. 51.6 μg/mL) and human liver cancer cells (HepG2) (IC_50_ values 23.4 vs. 53.2 μg/mL). With the same aim, very recently, the same group also designed the lipopeptide C_18_H_5_R_7_RGDS (LP) able, at the same time, to target tumors, to penetrate cells and to release drugs exploiting pH-sensitive properties [67]. The solubility of CUR in these vehicles was found higher than the previously reported peptide surfactant micelles, and the encapsulation efficiency (E.E.) and drug loading (D.L.) efficiency are (24.03 ± 0.16)% and (5.86 ± 0.04)%, respectively. The E.E. is lower than previously reported monomethyl poly(ethylene glycol)-poly(ε-caprolactone)-poly (trimethylene carbonate) (MPEG-P(CL-*co*-TMC)) copolymeric micelles (96.08%) [68] or spongosome and cubosome lipid NPs (99%) [69]. The D.L. is lower than polymeric micelles (14.07), but it is higher than cubosomes (2.7%). In vitro assays demonstrated a much higher cell proliferation inhibition on HepG2 cells and a lower cytotoxicity on L02 normal cells of CUR micelles compared to free CUR.

Another example reported in the literature is represented by two amphiphilic unimers containing an aliphatic hydrophobic chain (PDA) with two triple bonds and hydrophilic heads presenting the chelating agent DTPAGlu and the CCK8 bioactive peptide, respectively [70]. Aliphatic tails polymerized by UV after mixing the two unimers in a ratio of 70:30 produced elongated micelles with a relaxivity value of around 12 mM^−1^s^−1^. The same tail was functionalized with an RGD peptide to target integrin receptors [71].

Yao et al. achieved micelles in sub-30 nm size loading with camptothecin (CPT). The drug release pathway was actively mediated by RGD motifs targeting the ανβ3 integrin receptor, enhancing the therapeutic efficacy by an order of magnitude compared to free CPT against ovarian cancer cells.

The pituitary adenylate cyclase activating polypeptide (PACAP38) coupled to a docosahexaenoic acid (DHA: an ω-3 polyunsaturated fatty acid (PUFA)) was designed towards the creation of compartmentalized liquid crystalline assemblies of neuroprotective compounds [72]. The hormone PACAP38 is a ligand of the class B PAC1 GPCR, with intriguing compartmentalized architectures (vesicles embedding pep-lipid micelles and pep-lipid cubosomes with functionalized lipid bilayer interfaces) that are expected to increase the bioavailability of the studied class-B GPCR ligand.

Table 1 reports the peptide surfactant micelles list.

## 4. Polymeric Micelles

Polymeric micelles are self-assembled core–shell nanostructures formed in an aqueous solution consisting of amphiphilic block copolymers [73]. Polymeric micelles often have a lower CMC value compared to micelles prepared from conventional surfactants. In some cases, amphiphilic copolymers have CMC values lower than 10^−6^ mol·L^−1^, about two orders of magnitude lower than a non-ionic surfactant such as Tween 80. A crucial parameter for the process of self-assembly is the length of the hydrophilic and/or hydrophobic block. A high molecular weight of hydrophilic moieties aids the solubility of polymers in water as unimers, while molecules with a very long hydrophobic block tend to assemble by forming structures such as rods and lamellae. Monomer units can be arranged into two conjugated blocks each consisting of monomers of the same type (A-B-type copolymers), or they can form alternating blocks with different hydrophobicity (A-B-A-type copolymers). Poly(ethylene glycol) (PEG), with a molecular weight from 1 to 15 kDa, is the most present hydrophilic block in micelle-forming unimers. This molecule is largely employed due to its low toxicity and low cost, and it has been approved for injection applications by regulatory agencies. As alternative polymers, chitosan or poly(*N*-vinyl-2-pyrrolidone) (PVP) are also employed. At the same time, a variety of monomers may be used to build hydrophobic core-forming blocks: propylene oxide, l-lysine, aspartic acid, β-benzoyl-l-aspartate, γ-benzyl-l-glutamate, caprolactone (PCL), d,l-lactic acid (PLA) and some others. In some cases, phospholipid residues, homing two extremely hydrophobic long-chain fatty acyl groups, can also be successfully linked as hydrophobic core-forming groups. The contribution of hydrophobic interactions of carbon chains may considerably increase the stability of nanoaggregates compared with conventional amphiphilic polymer micelles.

### 4.1. PEG-PLA Micelles

**Figure 4:**
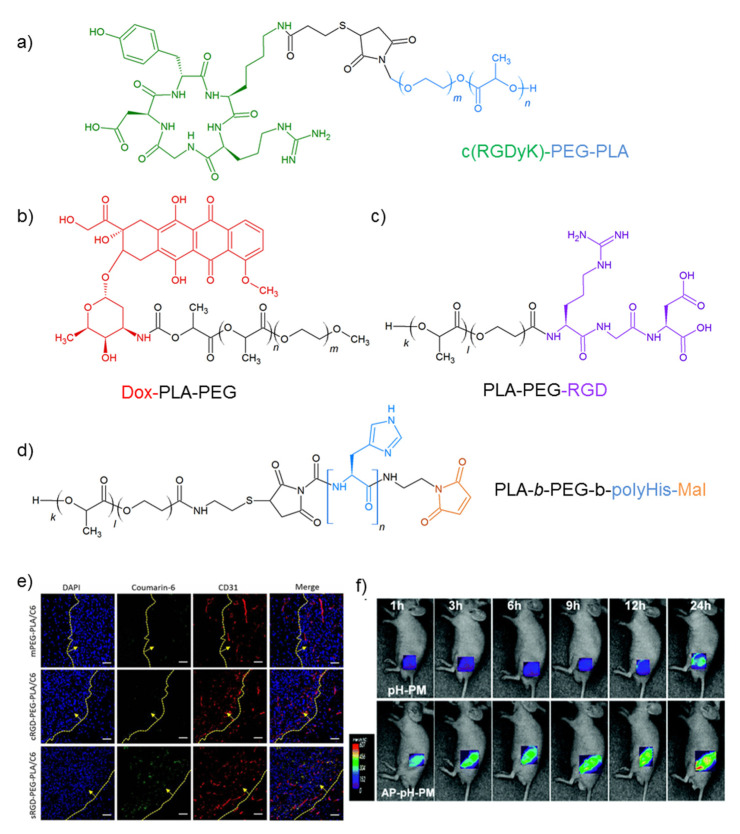
Structures of some PEG-PLGA micelle constituents (**a**–**d**); (**e**) distribution of different PLA micelles in glioma-bearing brain of nude mice (2 h and scale bar = 50 μm). The yellow lines are glioma margins, and the arrow points to the side of glioma. Blue, DAPI-marked cell nuclei; green, fluorescein-marked micelles; red, CD31-marked tumor microvessels (reprinted with permission from [78], Copyright 2017 American Chemical Society); (**f**) in vivo fluorescence images of MDA-MB-231 tumor xenografted nude mice treated with TRITC-pH-PM (upper row) and TRITC-AP-pH-PM (lower row) (reprinted with permission from [81], Copyright 2010 American Chemical Society).

Due to its good biodegradation and biocompatibility, the amphiphilic polymer PLA (PLG, also known as poly(lactic-*co*-glycolic acid), approved by the Food and Drug Administration (FDA)), has been well studied. Using different chemical approaches, PLGA was also efficiently decorated with PEG polymers, obtaining the conjugate PEG-PLGA. Therefore, PEG-PLA micelles were used to deliver hydrophobic drugs and contrast agents. cRGD-grafted PEG-PLA was developed in 2006 by Nasongkla et al. [74]. They loaded DOX and a cluster of super paramagnetic iron oxides (SPIOs) acting as a theranostic system in the micelle inner core. Rapid integrin receptor-mediated endocytosis facilitated the internalization of cRGD-encoded micelles inside SLK endothelial cells. Selective internalization was demonstrated by preventively treating cells with cRGD, which reduces the cytotoxic effects. PEG-PLA aggregates with different molecular weights were studied by Wang et al., grafting micelles with an RGD sequence [75]. Nanosystems were loaded with the antivascular drug CA4 with maximal entrapment efficacy in MPEG2000-PLA2000 micelles (98.14%). After demonstration of the selective uptake towards the endothelial cells (human umbilical vein endothelial cells (HUVEC) cells), the anti-proliferative activity tests pointed out the lower IC_50_ value of CA4 loaded in targeted micelles than in non-targeted micelles (IC_50_ = 0.52 ± 0.11 μM vs. 1.13 ± 0.18 μM). Another chemotherapeutic delivered by c(RGDfK)-PEG-PLA toward C6 glioblastoma cells was daunorubicin [76]. PEG-PLA-Dau micelles were prepared by co-assembly of two amphiphilic block copolymers (PEG-PLA-Dau and cRGD PEG-PLA). Enhanced uptake of cRGD-grafted micelles by C6 cells was observed via flow cytometry experiments, and they showed higher cytotoxicity than undecorated micelles. Poly(ethylene glycol)-co-poly(lactic acid) micelles functionalized with c(RGDyK) (Figure 4a) can reach glioblastoma multiforme tumor cells (GBM). GBM is angiogenesis-dependent, and integrin ανβ3 is widely overexpressed on tumor neovasculature, allowing it to overcome the blood–brain barrier (BBB). Lu et al. efficiently loaded these micelles, having a mean diameter of 35 nm, with PTX [77]. In vitro cytotoxicity studies showed that the presence of c(RGDyK) was able to enhance the anti-glioblastoma cell cytotoxic efficacy by 2.5-fold. In vivo assays indicated a significantly longer survival (48 days) of mice treated with c(RGDyK)-PEG-PLA-PTX micelles than mice treated with untargeted PEG-PLA-PTX micelles (41.5 days), free Taxol (38.5 days) or saline (34 days) with grafted RGD. The same group later, inspired by the peptide stapling technique, introduced two *N*-α-Fmoc-protected cross-linking alkene-containing amino acids, R8 and S5. At the end of a linear RGD, they synthesized a cyclic stapled RGD (sRGD) by the Grubbs ruthenium catalyst-mediated chemical strategy, expecting to endow it with the ability of BBB penetration [78]. A GSSG linker in the stapled RGD peptide sequence was introduced to achieve the mobility of the functional sRGD moiety while connecting to Mal-PEG3000-PLA2000. PTX-loaded sRGD-modified micelles in vivo could not only efficiently transverse the intact BBB of normal mice but could specifically target glioma cells of intracranial glioma-bearing nude mice.

PEG-PLA micelles were used to deliver, at same time, two anticancer drugs: DOX and CA4, in a multifunctional approach [79]. Wang et al. linked DOX to a polilactide moiety and an RGD sequence to PEG (Figure 4b,c) in order to expose the peptide to receptors overexpressed in actively proliferating endothelial cells and malignant tumor cells within a solid tumor. CA4-loaded micelles obtained by mixing the two copolymers were characterized by TEM, showing a uniform spherical structure and a mean diameter of 30 nm. These systems were tested in vitro and in vivo on B16-F10 tumor cells. Treatment with RGD-PEG-PLA-DOX-CA4 caused a distinctly superior outcome than treatment of single drug-loaded aggregates. CA4 kills the tumor vasculature directly and the corresponding cancer cells. Indirectly, the sustained release of DOX causes the death of the rest of the tumor cells.

PEG-PLA micelles were conjugated with peptides such as tLyp-1 peptide as a dual targeting ligand via a maleimide−thiol coupling reaction [80]. The decorated PTX-loaded micelles achieved a significant uptake on both HUVEC and C6 glioma cells due to the well-noted overexpression of NPR on these cells. Moreover, in vivo assays for tLyp-1-NP-PTX treatment prolonged the survival of mice bearing intracranial C6 glioma as compared to NP-PTX and Taxol (medium survival time: 37 d vs. 28 and 23 d, respectively).

Wu et al. coupled the maleimide-PEG-PLA block copolymer with thiolated AP peptide (CRKRLDRN) with specific binding affinity to IL-4 receptors of atherosclerotic plaques and breast tumor tissues [81]. The authors formulated DOX-loaded pH-sensitive micelles (DOX-AP-pH-PM) mixed with (MPEG)-poly(β-amino ester) (PAE) able to induce pH-sensitive drug release. In vivo studies showed that DOX-AP-pH-PM resulted in a 2-fold higher fluorescence intensity than DOX-pH-PMs in MDA-MB231 breast cancer tumor cells of mice and significantly superior tumor inhibition than free DOX and DOX-pH-PMs.

In order to target SSTR2 receptors, PEG-PLA micelles were also decorated with the octreotide peptide sequence. The resulting micelles were loaded with fluorescent probe DiI or DTX [82]. These nanoaggregates show a mean diameter of less than 80 nm, with a spherical shape and a high encapsulation efficiency. Flow cytometry and confocal microscopy results showed that OCT-PM-DiI enhanced the intracellular delivery efficiency via receptor-mediated endocytosis in human lung cancer NCI-H446 cells. Higher retardation of tumor growth after intravenous injections into a xenograft NCI-H446 tumor model was achieved in vivo, reducing the systemic toxicity. The same drug was also loaded in NGR-functionalized micelles [83]. The Asn–Gly–Arg (NGR) motif that facilitates the uptake of micelles by CD13-overexpressed tumor cells (fibrosarcoma, HT1080) and HUVEC in BALB/c mice bearing HT1080 tumor xenografts showed a stronger efficacy and less body weight changes in the NGR–PM-DTX group.

Lee et al. developed polymeric micelles using maleimide reactivity (Figure 4d) and constituted, from two block copolymers of poly(l-lactic acid)-b-poly(ethylene glycol)-b-poly(l-histidine)-TAT and poly(l-histidine)-b-poly(ethylene glycol), a pH-sensitive molecular chain actuator [84]. Micelles become physically destabilized at pH < 6.5, enhancing drug release, disrupting the cell endosomal membrane and increasing the intracellular drug concentration. DOX-encapsulated micelles were tested in many wild and multidrug-resistant (MDR) cell lines displaying a 3.8–8.8-fold lower IC_50_ than free DOX. Some representative in vitro and in vivo results are reported in Figure 4e,f. Xenograft tumors of human ovarian tumor drug-resistant A2780/AD, human breast tumor drug-sensitive MCF-7, human lung tumor A549 and human epidermoid tumor KB in a nude mice model all significantly regressed in size compared to free DOX, pH-insensitive micelles and pH-insensitive micelles with TAT exposed. With the aim to protect the TAT-PEG-DSPE amphiphilic peptide from proteolysis during the circulation in vivo, Wang et al. designed poly(l-glutamic acid)-b-poly(d,l-lactic acid) pGlu-PLA-based hybrid micelles for the intracellular delivery of DOX [85]. pGlu-PLA exhibited pH-responsive changes in conformation, which controlled the varied functionalities of micelles. Under tumor-acidic conditions, polymers with shorter poly(l-glutamic acid) blocks underwent a conformational change to form channels that accelerated the release of DOX. The conformational change also exposed TAT, promoting the cellular internalization of the micelles. In vivo studies demonstrated that DOX-loaded micelles with shorter poly(l-glutamic acid) blocks could efficiently gather in tumor tissues, restrained melanoma tumor growth and preserved the body weight. Micelles with longer poly(l-glutamic acid) blocks did not undergo the conformational change under acidic conditions, and they achieved weak in vitro and in vivo valuations.

### 4.2. PEG-PCL

**Figure 5:**
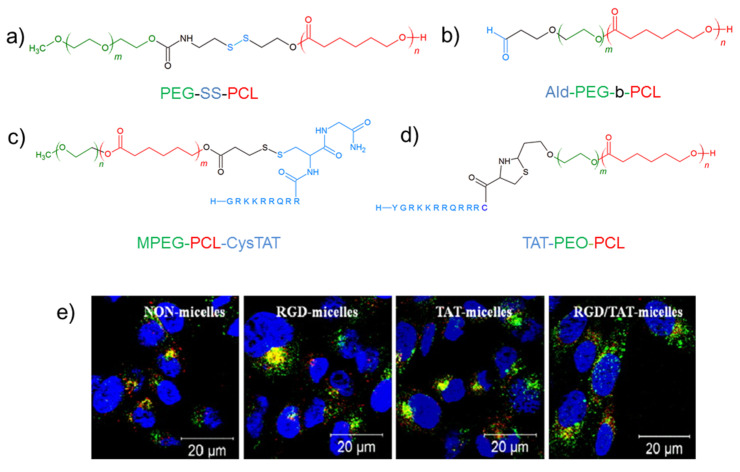
Chemical representation of some reported modified PEG-PCL polymers (**a**–**d**); (**e**) in vitro cellular distribution of the FAM-siRNA micellar formulations. FAM-siRNA was complexed in NON, RGD, TAT and RGD/TAT micelles. The incubation was with MDA435/LCC6-resistant cells for 4 h (siRNA 200 nm). The endosomes/lysosomes and nucleus were then stained with LysoTracker^®^ (red) and DAPI (blue), respectively (reproduced with permission from [106]).

Due to its desirable hydrophobicity that facilitates aggregation propensity and its low glass transition temperature that guarantees a partial rubbery core, poly(ε-caprolactone) (PCL) has been frequently used to form the core of self-assembled amphiphilic copolymers. DOX-loaded cyclic RGD peptide-functionalized biodegradable (PEG-PCL) micelles were developed to target glioma [86]. Zhu et al. introduced a disulfide bridge between hydrophilic and hydrophobic blocks (PEG-SS-PCL, Figure 5a) that significantly enhances both the intracellular drug release and in vitro antitumor efficacy of biodegradable micellar drugs. cRGD/PEG-SS-PCL micelles displayed a diameter of ca. 61 nm and a DOX loading of 14.9 wt% and triggered drug release in a reductive environment (10 mM glutathione). In vivo imaging and biodistribution studies in U87MG glioma xenografts revealed that mixed cRGD-PEG-SS-PCL/PEG-SS-PCL (molar ratios 20–80) micelles (cRGD20/PEG-SS-PCL) are efficiently accumulated, producing a fast drug release in cancer cells. DOX-loaded cRGD20/PEG-SS-PCL micelles presented minor side effects and superior tumor growth inhibition as compared to reduction-insensitive cRGD20/PEG-PCL and to non-targeting PEG-SS-PCL. In this context, the same group also developed micelles with a cross-linking core [87].

Dithiolane-functionalized trimethylene carbonate (DTC) was incorporated into a hydrophobic block cross-linking the core, inducing a fast redox-responsive drug release superior to PEG-PCL micelles. The cRGD PEG-DTC-PCL micelles exhibited a small hydrodynamic radius of ~50 nm, a high DOX loading capacity (18 wt%) and a high stability with low drug leakage. Mice treated with these micelles increased the median survival times two or three times with respect to the population treated with non-target micelles or with the free drug, respectively. PCL has been frequently used to form the core of self-assembled amphiphilic copolymers due to its desirable hydrophobicity that facilitates aggregation propensity and its low glass transition temperature that guarantees a partial rubbery core. The same polymer has been derivatized introducing an aromatic group as a side chain that confers more thermodynamic stability to the micelles (PEG-b-PBCL) than the parent PEG-b-PCL micelles. Both copolymers were decorated with either c(RGDfK) or p160, a cancer cell-specific peptide ligand [88]. PEG-b-PBCL-functionalized micelles show a 30% average major size of PEG-b-PCL micelles and better PTX encapsulation efficacy. In general, peptide decoration enhanced the selective cytotoxicity of encapsulated PTX against MDA-MB-435 cells over normal HUVEC and MCF10A cells; however, p160 micelles showed better binding and internalization in MDA-MB-435 cells than c(RGDfK) micelles. Successively, the same group grafted these copolymers via aldehyde conjugation (Figure 5b) with two peptides named 11 (RGDPAYQGRFL) and 18 (WXEAAYQRFL) [89]. Both peptides display better targeting affinity than previously reported cancer-targeting peptides p160 (VPWXEPAYQRFL) and cyclic RGDfK [90]. Functionalized micelles loaded with lipophilic cyanine fluorescent probe DiI showed higher cellular uptake in MDA-MB-435, MDA-MB-231 and MCF7 cell lines using flow cytometry and confocal microscopy techniques than p160, c-RGD-modified or naked micelles. The same authors [91] also reported on the development of multiple functions integrated into one system, including the ability to entrap a combination of therapeutic entities with different physicochemical properties (i.e., small interfering RNAs, siRNA and DOX), passive and active cancer targeting mediated by the Tat sequence and RGD4C, respectively, cell membrane translocation and pH-triggered drug release. All set micelles have an adaptable core that is able to stably complex siRNA and conjugate DOX. In addition, they take in a virus-like shell for cell-specific recognition and efficient cellular uptake. The authors highlight that these micelles may well release DOX by a pH-triggered mechanism via a hydrazone bond. Peptide-functionalized micelles, mainly RGD/TAT micelles containing mdr1-siRNA and DOX, demonstrated significant cellular uptake, enhanced DOX penetration into nuclei and, lastly, improved DOX cytotoxicity in DOX-resistant cells. These data revealed the remarkable potentiality of this multifunctional micellar nanomedicine both for proficient delivery of anticancer drugs and oncogene-silencing siRNA to their cellular and molecular targets. This system enhanced the efficacy of DOX in multidrug-resistant MDA-MB-435 human tumor models that overexpress P-glycoprotein (P-gp), through coincident delivery of DOX and siRNA against P-gp expression.

Moreover, PEG-b-PCL was grafted with octreotide by Zhang et al. [92]. Receptor endocytosis was demonstrated by adding free octreotide that inhibited the cellular uptake of Oct-decorated micelles. PTX entrapped (E.E. > 90%) in the labeled aggregates, with an average diameter of 30 nm, and salinomycin (SAL) loaded in PEG-b-PCL were used in combination therapy to eradicate breast cancer. In in vivo assays, this therapy with Oct-M-PTX plus M-SAL produced the highest efficacy in the MCF-7 xenografts, in agreement with the combination treatment in vitro. The same group built similar micelles to target SSTR2 with lanreotide, another somatostatin analogue [93]. Specific cell uptake and cytotoxicity were demonstrated on two cancer cell lines (H446 and MCF-7 cells) with different expression levels of SSTR2. Furthermore, treatment with lanreotide-PM-PTX micelles produced a stronger tumor inhibition in vivo test as compared with the control groups on nude mice with both H446 and MCF-7. Moreover, minor side effects such as less body weight loss, lower hemolysis and lower myelosuppression were also observed.

Gu et al. designed PEG-PCL micelles conjugated to an activable low-molecular weight protamine (ALMWP), containing a sequence of polycationic CPP, a matrix metalloproteinase (MMP)-sensitive peptide linker and a polyanionic inhibitory domain [94]. In in vitro assays, ALMWP-NP exhibited significantly elevated MMP-dependent cellular accumulation in C6 cells via lipid raft-mediated endocytosis and energy-dependent macropinocytosis and enhanced the cytotoxicity of PTX. In vivo imaging and glioma distribution justified its specific accumulation in the glioma. Improved glioma targeting and tumor penetration were demonstrated in nude mice bearing intracranial C6 glioma. Animals treated with ALMWP-NP-PTX survived longer than those treated with saline, free Taxol^®^, NP-PTX and LMWP-NP-PTX.

Tanaka et al. developed a carrier for the systemic delivery of a non-viral gene vector based on methoxypoly(ethylene glycol) (MPEG)/PCL diblock copolymers conjugated with a Tat analogue through an ester or disulfide linkage [95] (Figure 5c). Sizes of MPEG-PCL micelles were larger than those of Tat-functionalized nanoparticles, and the zeta potential of MPEG-PCL showed a negative charge, whereas that of Tat analogue-conjugated MPEG-PCL showed a positive charge.

In vitro transfection experiments in COS7 cells indicated that the luciferase activity was higher with pCMV-Luc with MPEG-PCL-ester-Tat or MPEG-PCL-SS-Tat than that with naked pDNA. MPEG-PCL-SS-Tat greatly increased the transfection efficiency compared to MPEG-PCL-ester-Tat in COS7 and S-180 cells due to ability to release the payload in a reductive environment. The same carrier was used later to deliver siVEGF [96]. The cellular uptake ability after transfection with fluorescent 6-carboxyfluorescein-aminohexylphosphoramidite (FAM)–siRNA with MPEG–PCL-SS-Tat was significantly higher than that with FAM–siRNA only. Tumor suppression was observed when treating S-180 tumor-bearing mice with MPEG–PCL-SS-Tat/si VEGF complexes compared with naked siVEGF and MPEG–PCL-SS-Tat/si control treatment. This result is attributed to the suppression of angiogenesis in the tumor tissue in a highly sequence-specific manner, as demonstrated by evaluating the expression level of CD31. The same group designed a new cytoplasm-responsive gene carrier peptide, stearoyl (STR)-CH_2_R_4_H_2_C [97]. This peptide contains: (i) four Arg residues promoting endocytosis of SiRNA, (ii) two His residues inducing the early endosomal escape by the proton sponge effect and (iii) two Cys residues that protect nucleic acids against nuclease, forming an intermolecular disulfide cross-linkage in non-reducing environments, such as the extracellular space and blood. The siVEGF-loaded MPEG–PCL–CH_2_R_4_H_2_C reached a significantly greater silencing effect than free siVEGF with low cytotoxicity and, when tested in vivo, significantly inhibited tumor growth in S-180 tumor-bearing mice. The same carrier was recently loaded with anti-RelA siRNA, which is a subunit of NF-κB. It inhibited metastasis and it showed an inhibitory effect in a lung metastasis mouse model [98].

The employment of PEG-b-PCL nano-sized micelles modified with CPPs can facilitate direct, intranasal brain delivery [99]. Kanazawa et al. linked the Tat analogue through an ester bond to MPEG-PCL, obtaining nanoparticles with a lower diameter compared to undecorated micelles (73 nm vs. 116 nm) and a positively charged corona (zeta potential 6.0). Application of MPEG-PCL-Tat micelles can facilitate direct, intranasal brain delivery and prolong the survival time of rats with intracranial C6 glioma.

Later, the same authors extended the novel, noninvasive, efficient and safe therapeutic strategy to malignant glioma delivering siRNA/CPT [100]. The intracellular delivery and antitumor effects of siRNA for Raf-1, which plays a role in cell proliferation and apoptosis, in C6 rat glioma cells were explored. In detail, the authors assessed the therapeutic effects in rats with malignant glioma treated using the co-delivery of siRNA/CPT, detecting a consistent increase in the mean survival time compared to single-therapeutic delivery (28 d vs. 20 d).

This carrier can find different applications by exploiting its ability to cross the BBB [101]. The treatment of intractable brain diseases such as cerebral ischemic reperfusion injury may well facilitate delivering anti-TNF-α siRNA, which has an anti-inflammatory effect. The N/P ratio was increased from 5 to 30, inducing a relative decrease in fluorescence and in the particle size from 100 to 50 nm. In order to assess its effectiveness, PEG-PCL-Tat/anti-TNF-α siRNA was administrated in t-MCAO model rats, measuring the infarcted area compared to anti-TNF-α siRNA and the empty carrier. Results displayed a reduction in this area and TNF-α production, suppression of brain weight and worsening of the neurology score for the PEG-PCL-Tat/anti-TNF-α group because siRNA-only is unable to penetrate the nasal mucosa and has poor in vivo stability. However, improvements are still necessary to selectively deliver drugs to tumor cells and penetrate the brain, avoiding side effects to normal brain tissue. Kanazawa et al. [102] formulated tumor-selective novel mixed micelles consisting of Tat-conjugated polymer micelles and stearoyl-modified bombesin (Bom/PEG-PCL-Tat). The dual system can exploit the activity of 7–14 bombesin (Bom), which has displayed, with its analogue derivatives, active targeting properties [103,104]. 7–14 Bom is the truncated sequence of the wildtype endogenous sequence that is able to recognize GRPR receptors, overexpressed in many tumors such as prostate cancer and ovarian, but also in C6 glioma cells. Bom/PEG-PCL-Tat micelles were selectively internalized at a high rate by GRPR-positive C6 glioma cells, whereas superior accumulation in GRPR-negative COS7 cells was not observed. The presence of two peptides implicated two cellular uptake pathways in this process: macropinocytosis, due to interactions with Tat, and clathrin-mediated endocytosis, induced by bombesin. Furthermore, CPT-loaded Bom/PEG-PCL-Tat micelles increased their cytotoxic effects against C6 glioma cells compared to micelles lacking bombesin. In vivo assays on C6 glioma orthotropic grafted ratsBom/PEG-PCL-Tat mixed micelles exhibited site specificity and superior therapeutic effectiveness, although the bombesin inclusion here did not produce therapeutic effects commensurate with complete remission.

Xiong et al. designed a novel family of biodegradable (PEO-b-PCL)-based copolymers containing polycationic side chains on the PCL block, i.e., PEO-b-PCL with spermine (PEO-b-P(CL-g-SP)), which contains two amino groups and two imino groups and is produced via enzymatic catalysis in living organisms, tetraethylenepentamine (PEO-b-P(CL-g-TP)) or *N*,*N*-dimethyldipropylenetriamine (PEO-b-P(CL-g-DP)) [105]. These polymeric micelles were able to bind siRNAs and behaved as safe and effective carriers for delivery and efficient uptake through endocytosis by MDA435/LCC6 cells transfected with MDR-1, which encodes for the expression of P-glycoprotein (P-gp). On the basis of these results, later, the authors decorated the micelles with integrin ανβ3-targeting peptide (RGD4C) and/or Tat (Figure 5d) [106]. The presence of both peptides increases the cellular uptake compared to unlabeled micelles (Figure 5e). Moreover, it silenced P-gp expression and increased the DOX concentration in the cytoplasm and nucleus of resistant MDA435/LCC6 cells. This system has evolved to integrate multiple functions of passive and active cancer targeting mediated by the Tat sequence and RGD, respectively, cell membrane translocation and pH-triggered drug release [91]. Moreover, the authors highlight that these micelles may well release DOX by a pH-triggered mechanism. This system enhanced the efficacy of DOX in multidrug-resistant MDA-MB-435 human tumor models that overexpress P-glycoprotein (P-gp), through coincident delivery of DOX and siRNA against P-gp expression. All of the data reveal the remarkable potential of this multifunctional micellar nanomedicine both for proficient delivery of anticancer drugs and oncogene-silencing siRNA to their cellular and molecular targets.

Inspired by these results, Zhu et al. designed cRGD- and TAT-modified cross-linkable micelles (cRGD/TAT CMs), in which the TAT peptide was shielded by relatively long PEG chains [107]. Mixed micelles with molar ratios of 20% cRGD and 10% TAT were tested on ανβ3-overexpressing U87MG glioma cells. In flow cytometry assays, the selectivity of cRGD/TAT CMs was observed with an 8.3-fold and 18.3-fold higher uptake than cRGD20 CMs and PEG CMs, respectively, and a stronger cytotoxic effect after loading with DTX was detected. In vivo cRGD20/TAT10 CMs, probably via receptor-mediated endocytosis and deep tumor penetration, induced the highest tumor DTX level (8.6% ID/g) and minor side effects and inhibited tumor growth. Very recently, Zhang et al. described a self-assembled PEO-b-P(CL-g-SP) ligand, decorated with a dual bioactive sequence to deliver SiRNA and DTX to prostate cancer (PCa) cells [108]. Particles were grafted with *N*-[*N*-[(*S*)-1,3-dicarboxypropyl] carbamoyl]-(*S*)-lysine (DCL) in order to bind, via strong hydrogen bonds, prostate-specific membrane antigen (PSMA), which is a *trans*membrane glycoprotein that is overexpressed by 100–1000 times in PCa cells by means of a further increased expression in metastatic and castration-resistant carcinomas [109]. The next bioactive molecule is a TAT sequence which can enhance the cell penetration effect. Target micelles self-assembled and entrapped DTX and anti-NS siRNA to exactly affect the mitotic process of tumor cells. In in vitro assays, chemotherapy drugs blocked apoptosis, but targeted micelles with dual drugs significantly enhanced the effect. Depending on the downregulation of the NS gene and protein expression, this system inhibited the G1/S and G2/M mitotic phases.

### 4.3. PEG-Poly(l-amino Acid)

**Figure 6:**
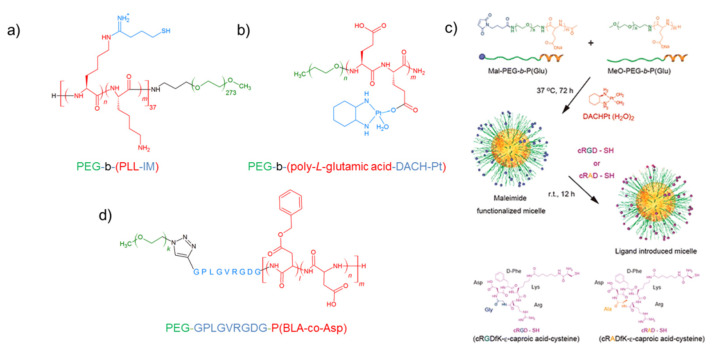
(**a**,**b**,**d**) Chemical formulas for some selected PEG-Poly(l-amino acid) micelle unimers; (**c**) scheme for the design and synthesis approach for ligand-linked DACHPt-containing micelles (reproduced with permission from [116]).

Polyion complex (PIC) micelles are composed of charged block water-soluble copolymers, and they have a narrow size distribution. The first PIC micelle formation was observed with the pair of copolymers poly(ethylene glycol)-block-poly(l-lysine) (PEG-b-PLL) and PEG-b-poly(α,β-aspartic acid). In the last decade, a number of PICs have been investigated as carriers of small interfering RNA (siRNA). These short anionic synthetic molecules can be adsorbed on the PICs through electrostatic interactions. SiRNAs induce highly efficient gene silencing at the translation level; therefore, they can be a useful tool as a therapeutic agent in tumor care. Matsumoto et al. selected PEG-b-poly(l-lysine) to deliver SiRNA [110]. Core–shell-type PIC micelles with a disulfide cross-linked core were prepared through the assembly of iminothiolane-modified PEG-b-(PLL-IM), (Figure 6a) and siRNA. This modification allows achieving major stability of the aggregates with enhanced van der Waals and dipole–dipole interactions as well as hydrogen bonding capability by siRNA or other polymer chains. Moreover, in the presence of glutathione in reductive environments of cancer cells, the micelle can release the siRNA. Later, the same authors functionalized the same copolymer with cRGD [111]. cRGD was anchored by thiazolidine ring formation between the acetal function present on the hydrophilic segment and the *N*-terminal cysteine capronic acid residue-containing peptide. These micelles showed an average diameter of 45 nm, with a narrow distribution. cRGD-grafted micelles were found to be able to increase the gene silencing ability, improving cell uptake in an in vitro experiment on a HeLa cell line. After intravenous injection into mice, the same formulation also improved accumulation in both tumor mass and tumor-associated blood vessels. These systems were successively improved by functionalizing the copolymer with dithiobispropionimidate (DTBP) and by encapsulating cholesterol-conjugated siRNA (Chlo-RNA) [112]. Chemical physical characterizations confirm that the Chol moiety did not alter the micelle formation behavior. Stability, induced by Chol’s presence, was demonstrated comparing the resistance to both counter polyion exchange with dextran sulfate and the dilution in serum-containing medium. cRGD-decorated micelles’ surface enhanced the in vitro gene silencing activity of siRNA regardless of the Chol presence. Both results were confirmed by in vivo experiments. The cRGD moiety significantly aided accumulation of siRNA in a subcutaneous cervical cancer model. Therefore, a significant sequence-specific gene silencing was observed, exploiting the synergistic effect of active target ability and improved stability.

In the last decade, camptothecin (CPT) was also encapsulated in many delivery systems. However, poor results were obtained, related to the low loading efficiency and side effects, including inflammation or a phylaxis induced by the acidic degradation of polymers. Guoet et al. synthesized cationic polypeptide poly-lysine-block-poly-leucine (PLys-b-PLeu) via the ring-opening polymerization of *N*-α-carbobenzoxy-l-lysine and l-leucine (Leu) and further grafted it with PEG and an RGD peptide [113]. The drug was entrapped as dimer DCPT, obtained by reacting CPT and 2-hydroxyethyl disulfide. Stable DCPT-loaded RGD-PEG-p(Lys)-p(Leu)(DRPPP) micelles displayed a high encapsulation efficiency of 89.7% and a high D.L. capacity of 46.1% compared to micelles lacking a PEG segment which reduces the zeta potential. Active targeting was verified on MDA-MB-231 (with higher receptor overexpression) and MCF-7 cells. The drug produced an enhanced cytotoxicity against MDA-MB-231 cells compared to MCF-7 cells, inducing cell apoptosis. Han et al. designed a double pH-sensitive copolymer based on the same copolymers labeled with the Tat peptide [114]. Lys residues of Tat and polyLys peptides were modified into two different β-carboxylic amides. The amides were demonstrated to undergo stepwise hydrolyzation, responding to acidic tumor extracellular environments (pH ≈ 6.5) and more acidic cell endo/lysosomes (pH ≈ 5.0), by which the amino groups and their original functions were recovered. Overall, these double amidation charge reversal strategies help to realize tumor targeting uptake and nuclear delivery simultaneously.

Polymeric micelles can prolong the blood circulation and favor the tumor accumulation of oxaliplatin. Dichloro(1,2-diaminocyclohexane)platinum(II) (DACH-Pt), the oxaliplatin parent complex, can be loaded in micelles to exploit the ability to exchange the chlorine ligand with nucleophiles such as the carboxilic group on the side of poly-l-glutamic acid (Figure 6b). Cabral et al. prepared stable micelles through polymer–metal complex formation of DACHPt with poly(ethylene glycol)–poly(glutamic acid) block copolymer [PEG–P(Glu)] [115]. Later, they decorated the PEG strands in the outer shell layer of the same preparation with various cRGD conjugation ratios, ranging from 5% to 40% (see Figure 6c). This modification allows micelles to overcome the BBB and reach the glioblastoma (U87MG) [116]. Moreover, micelles were functionalized with cyclic-Arg-Ala-Asp(cRAD) peptides to compare the ability to drive the aggregates. All micelles display physicochemical parameters including their size, surface, charge and the amount of incorporated drugs suitable for their in vivo use. For cRGD-linked polymeric micelles (cRGD/m), after 3 h of incubation, the internalized platinum amount determined by ICP-MS was approximately 2.5-fold higher than that with 20% cRAD-linked DACHPt/m (20% cRAD/m). Implanted U87MG-Luc2 cancer cells into the brains of BALB/c nude mice were monitored by bioluminescence for 22 days, exhibiting a significant higher tumor growth inhibitory effect of 20% (cRGD/m) vs. 20% cRAD/m and oxaliplatin. The same micelles were tested in vivo during the treatment against lymph node metastasis (LNM) in the presence of the primary melanoma [117]. Both cRGD-DACHPt micelles and non-targeting MeO-DACHPt micelles exhibited comparable activity against primary tumors and the established metastatic foci in lymph nodes. However, decorated micelles significantly improved the efficacy against LNM, limiting cells’ spread from the primary tumor to lymph nodes. The effective inhibition of the diffusion of cancer cells is higher than that of non-target micelles, free cRGD and their combinations. The same group prepared similar micelles entrapping *cis* platin (CDDP) in order to eradicate head and neck squamous cell carcinoma (HNSCC) and cancer stem-like cell (CSC) sub-populations in the tumors, which are not sensitive to chemotherapy [118]. This evidence is in agreement with the cRGD peptide-decorated surface of CDDP/m, promoting the targeting of orthotopic HNSCC tumors bearing CSCs and their lymph node metastases, and improving micelles’ activity against differentiated CSCs and in HNSCC cells.

Song et al. designed amphiphilic polymers condensing side chain carboxylic groups of PolyGlu polymer with tocopherol as a hydrophobic moiety and PEG [119]. DTX and CDDP were encapsulated in PLG-g-Ve/PEG, exploiting hydrophobic interactions and the carboxylic chelating property, with a drug loading content (DLC) of 4.3% and 8.5%, respectively. Drug-loaded micelles decorated with cRGD exhibited a synergistic cytotoxic effect and an enhanced internalization rate in mouse melanoma (B16F10) cells. The tumor volume and the tumor growth rate (TGR%) at day 16 were reduced for mice treated with cRGD-M(DTX0.5/Pt) twice more than their counterpart treated with undecorated micelles. Moreover, the whole population was still alive 25 days after the first administration, whereas only less than 20% of the mice treated with M(DTX0.5/Pt) survived.

Guan et al. demonstrated that the binding of PEG-b-PGA to carboxylic group 1-(3-aminopropyl)imidazole (API) that mediates pH-responsive charge switching via protonation leads to enhanced drug accumulation in tumor cells [120]. Micelles obtained by mixing RGD–PEG-b-PLA and PEG–PGA–API in the ratio of 1:4 exhibited a hydrodynamic diameter of 30.1 nm, 4.42 mV of zeta potential and approximately 90% of drug encapsulation efficiency. Confocal laser scanning microscopy and flow cytometry experiments indicated that combining the capability of an RGD target and pH-sensitive charge switching significantly enhanced the cellular uptake of B16F10 cells overexpressing integrins. 3-(4,5-dimethylthiazol-2-yl)-2,5-diphenyltetrazolium bromide (MTT) assay ανβ3 also showed that our hybrid RGD-decorated micelles were much more cytotoxic to B16F10 cells than undecorated micelles.

cRGD was also exploited by Liu et al. to target glioma cells [121]. They formulated homogeneous polyionic micelles based on maleimide-PEG-g-*N*-diethanolamine (PDEA) and maleimide-PEG-g-poly(aspartamide) (PAsp), with an average diameter of 60 nm. The distribution of c(RGDfC) polyionic complex micelles was evaluated by the CdTe quantum dot marking technique. They demonstrated an inhibitory effect in in vivo experiments, exhibiting a statistical increase in the survival time of rats treated with c(RGDfC) polyionic complex micelles, compared with control groups.

The Kataoka group developed pH-sensitive micelles using acetal-poly(ethylene glycol)-b-poly(β-benzyl l-aspartate) (Acetal-PEG-b-PBLA) as a precursor [122]. After anchoring the cRGD by a thiazolidine ring to the PEG moiety, the hydrophobic polymer PBLA side chain was partially functionalized with hydrazinyl by an ester–amide exchange, inducing pH-sensitive hydrazone formation. cRGD-labeled epirubicin-loaded polymeric micelles (cRGD-Epi/m) achieved faster and higher penetration into U87MG cell-derived 3D spheroids than non-target micelles. In vivo, by monitoring the tumor growth by measuring the bioluminescence intensity, it was observed that cRGD-Epi/m significantly reduced the growth of the tumors, showing a 12-fold lower photon flux than the group treated with Epi/m on day 21 after the beginning of treatment.

Keet et al. designed an enzyme-responsive peptide-linked block copolymer, PEG-GPLGVRGDG-P(BLA-co-Asp) (Figure 6d), as an intelligent drug delivery vehicle for addressing doxorubicin (DOX) [123]. Block copolymers were achieved via the click reaction to bear peptide (alkynyl-GPLGVRGDG) on the end of PEG for initiating the ring-opening polymerization of β-benzyl l-aspartate *N*-carboxyanhydride (BLA-NCA) by terminal amino groups followed by partial hydrolysis of PBLA segments. MMP-2 triggered dePEGylation to remove PEG-GPLG by cleavage of the amido bond between G and V, and the residual RGD on the surface will act as a ligand to interact with the cell membrane to facilitate the cellular internalization. The micelle was able to entrap DOX efficiently through the synergistic effect of the benzyl group-based hydrophobic and carboxyl moiety-based electrostatic interactions. In in vitro assays against HT1080 cells overexpressing MMP-2, DOX-loaded micelles showed an approximately 4-fold increase in the cellular internalization amount as compared with free DOX, producing a half maximal inhibitory concentration (IC_50_) value of DOX-loaded micelles of 0.38 μg/mL compared with 0.66 μg/mL of free DOX due to MMP-triggered dePEGylation.

To improve drug loading properties and drug release, Qiu et al. synthesized the poly(ethylene glycol)-b-poly(α-aminopalmitic acid) (PEG-b-PAPA) block copolymer [124]. PEG-b-PAPA copolymers displayed excellent solubility in common organic solvents, and stable micelles were formed in aqueous solution at physiological pH, with a smaller size of 59 nm and a low CMC (2.38 mg/L). Lipophilic tails on side chains allowed entrapping hydrophobic drugs such as DTX due to the strong hydrophobic interactions and the enzymatic degradation, leading to a fast intracellular drug release. cRGD-PEG-b-PAPA produced a relevant anticancer effect (IC_50_ = 0.15 μg DTX equiv/mL) in in vitro tests on B16F10 cells. Excellent results were observed in in vivo therapy studies: DTX–cRGD-Lipep-Ms exhibited minor tumor growth of B16F10 melanoma, an improved survival rate (36 d vs. 9 d) and minor side effects such as weight loss as compared to free DTX. Successively, the same nanosystem was loaded with monomethyl auristatin E (MMAE), a peptide used in conjugation with an antibody in relapsed Hodgkin lymphoma and systemic anaplastic large cell lymphoma [125]. The peptide, through the strong intermolecular hydrogen bond and hydrophobic interaction, was entrapped with a loading content of 5.5 wt%, which was 55-fold higher than that of PEG-b-PLA micelles. MMAE-loaded cRGD-Lipep-Ms (MMAE-cRGD-LipepMs) at 37 °C were internalized by receptor-mediated endocytosis, enhancing the delivery compared to MMAE-LipepMs. Therapeutic studies using HCT-116 tumor-bearing mice show clearly that MMAE-cRGD-LipepMs induced major tumor inhibition (78%) compared to both free MMAE (26%) and MMAE-loaded Lipep-Ms (48%). Moreover, hematological parameters were in the normal range, reflecting reduced side effects.

### 4.4. Pluronics

Pluronics are a family of nonionic triblock copolymers, also known as poloxamers. They are shaped as poly(ethylene oxide)-b-poly(propylene oxide)-b-poly(ethylene oxide) types of block copolymers with different lengths, represented as PEO_m/2_-b-PPO_n_-b-PEO_m/2_, where m and n indicate the average number of repeat units. Poloxamers, also known by the trade names of Kolliphor^®^ (for the pharma grade) and Synperonic^®^, are composed of a central hydrophobic unit decorated by two hydrophilic chains. By varying the lengths of the chain segments, a significant difference in the values of CMC and E.E. can be achieved. The last parameter is a pivotal factor in the optimization of pharmaceutical formulations [126]. Pluronic micelles have many outstanding features that include a relatively low in vivo toxicity and an appropriate size that restricts renal excretion. Moreover, another important factor is overcoming drug resistance by the inhibiting the efflux action of P-glycoprotein. Pluronic micelles deliver drugs in a spatially and temporally controlled manner, with enhanced intracellular uptake via fluid-phase endocytosis rather than a passive diffusion, reducing their intracellular uptake by normal cells. This shielding effect allows decreasing the drug interaction with healthy tissues. Peptide functionalization allows expanding the application range.

Chen et al. decorated Pluronic^®^ F127 micelles with c(RGDyK) conjugated to the *N*-hydroxysuccinimide-activated PEO terminus [127]. Pluronic F127 was chosen to increase the in vitro stability of micelles compared to that composed of Pluronic P105 previously studied. Micelles loaded with DOX and PTX (RGD-PF-DP) for dual therapy showed a spherical shape, as determined by TEM, and an average size compatible with their use in vivo (23 nm). The dual-functional effect was assessed in both integrin-expressed angiogenic HUVEC and in vitro-based models. Functionalized micelles can enhance cellular uptake and tumor spheroid penetration, and the cell apoptosis activity in MDR-KBv cells. Furthermore, c(RGDyK)-FP-DP displayed stronger inhibition potency on the growth of KBv MDR tumor spheroids. The same authors tested these micelles to enhance blood–brain barrier penetration and improve drug accumulation via integrin-mediated transcytosis/endocytosis on glioma cells [128]. The anti-glioma effect of RGD-PF-DP in vivo was compared with the survival time of intracranial glioma-bearing mice. The median survival time of mice treated by RGD-PF-DP (39 days) was significantly longer than that of mice treated with saline (23 days), DOXPTX (23 days) and non-target PF-DP (31 days). Pluronic^®^ F127 was mixed with amphiphilic molecules functionalized with the D4 sequence (D4-(AdA)_2_(C18)_2_) to address active targeting towards EGFR-overexpressing receptors in cancer cells [129]. Mixed aggregates decorated with the D4 sequence and a scrambled sequence were achieved by means of adding equal percentages (2.5% *w*/*w*) to PF 127 and a modest mass amount (1‰) of Rhod-PE. The in vitro test investigated by fluorescence microscopy demonstrated that the cellular binding of D4 peptide-conjugated micelles was more efficient in PLC/PRF/5 cancer cells overexpressing EGFR than in HepG2 tumoral cells with low EGFR expression, whereas the same aggregates decorated with the scrambled sequence exhibited a lower uptake.

### 4.5. DSPE-PEG Micelles

**Figure 7:**
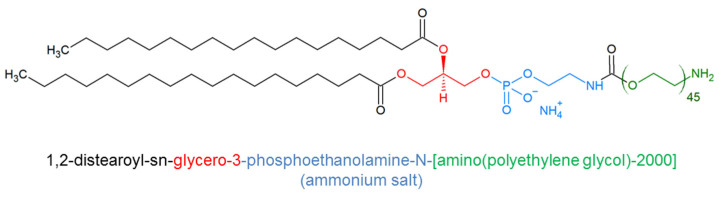
Chemical structure of DSPE-PEG(2000).

1,2-Distearoyl-sn-glycero-3-phosphorylethanolamine (DSPE) was proposed in its different PEGylated version (DSPE-PEG) (Figure 7) as a formulative tool of micelle preparation. Indeed, the PEGylated phospholipid self-assembles in micelles, providing the DSPE lipid tail a strong hydrophobic driving force. The length of PEG tunes the aggregates’ diameter and the solubility of hydrophobic drugs. Indeed, higher CMCs for a longer PEG chain length were observed. The bulky PEG(2000) head group imparts sufficient curvature to the core−corona interface to form oblate spheroidal micelles with a maximum diameter of 18 nm [130]. These observations push clinical applications due to their safety, biocompatibility and biodegradability; consequently, PEG-DSPE2000 is already approved as a component for human use in the liposomal marketed product Doxil^®^. The key advantage of this lipid-based nanocarrier is its simple preparation even at large scales, which allows an easy transition to clinical applications. Moreover, the PEG terminal can be additionally functionalized with a peptide sequence.

Zhang et al. stitched cRGDfK to DSPE-PEG, obtaining self-assembled micelles [131]. The presence of the peptide does not induce significant variations in the size (15 nm) and shape (spherical) of aggregates, although the zeta potential is more negative due to the presence of negative charges. These nanosystems were loaded with quercetin, a flavonoid able to induce apoptosis in tumor cells. The surface plasmon resonance (SPR) technique and real-time confocal analysis were employed to prove ανβ3 integrin-overexpressing B16 cells’ specific binding and uptake. In vivo, cRDGfK modification enhanced the therapeutic effects on B16 tumor-bearing mice and showed lower systemic and pulmonary toxicity compared with non-target micelles. The same aggregates have been modified by inserting a disulfide bridge between the hydrophilic component and the phospholipid, inducing a reduction process in a tumoral reductive environment [132]. Micelles were loaded with anlotinib, a novel small-molecule multi-target tyrosine kinase inhibitor, in order to achieve an E.E. of 98.64% and a D.L. of 8.98%. This drug is employed for the treatment of melanoma and lung metastases in oral administration with a short half-life and in body low blood concentrations. The encapsulation can improve this parameter, mitigating the side effect, prolonging the blood circulation time and increasing the effective absorption by tumor cells. These promising results were observed in B16F10 tumor-bearing mice.

DSPE-PEG was conjugated with other bioactive peptides as well as CCK8 [133]. Stable lamellar aggregates were obtained by self-assembling DSPE-PEG2000-CCK8 or mixing up to 50% with (C18)_2_DTPAGlu-Gd in order to formulate a diagnostic tool for MRI. By increasing the amount of (C18)_2_DTPAGlu-Gd lamellar aggregates in rod-like micelles, a transition was observed. Proton relaxivities for micelles were 17.2 M^−1^ms^−1^, although values were the result of different combinations of local and global contributions. The SSTR2 receptor was targeted by lanreotide-labeled micelles [134]. Zheng et al. reported the ability of these micelles in vitro and in vivo to reach tumors overexpressing SSTR2. This property was demonstrated previously in vitro and in vivo with lanreotide free peptide treatment. PTX entrapped with a very high E.E. (<95%) was found to be effective in retarding tumor growth compared with the data achieved for non-targeted micelles or for the free drug.

Furthermore, the major efficacy of PTX encapsulated in micelles for active targeting was demonstrated through grafting with the GE11 peptide able to address EGF receptors [135]. The average size of GE11-PEG-DSPE/paclitaxel micelles is larger than previous reported aggregates (35 ± 2.8 nm), and the E.E. was lower (74.1%). Hep-2 cell proliferation was 2.3 times more inhibited by GE11-PEG-DSPE/PTX micelles compared to mPEG-DSPE/paclitaxel micelles and Taxol in vitro. The same peptide was conjugated on the surface of phospholipid micelles through a peptide sequence matching the GGGSGGGSC linker to reduce the steric hindrance [136]. DOX was encapsulated with a DLC value of 15 wt%, with a drug loading efficiency (DLE) of 99%. To confirm the specific endocytosis of GE11 micelles through EGFR, a competition assay was conducted on SMMC-7721 cells with an excess of anti-EGFR monoclonal antibody C225 (cetuximab). In tumor-bearing nude mice, GE11-modified DiR-loaded micelles also exhibited a much higher level of DiR uptake in tumor tissue than non-targeted micelles according to NIRF imaging.

### 4.6. Others Micelles

Polymeric micelles can be formulated by choosing from the wide range of biocompatible functional hydrophobic polymers. Vene et al. selected two degradable hydrophobic copolymers: poly(ethylene glycol)-b-poly(benzyl malate) (PEG42-b-PMLABe73) and biotin-poly(ethylene glycol)b-poly(benzyl malate) (Biot-PEG62-b-PMLABe73) [137].

These copolymers belong to the poly(malic acid) (PMLA) family that represents biodegradable intomalic acids under physiological conditions [138]. The presence of biotin residues allows grafting the biotinylated cyclic RGD peptide via the bridging of streptavidin. Micelles designed from PEG42-b-PMLABe73, Biot-PEG62-b-PMLABe73 and RGDBiot-StreptBiot-PEG62-b-PMLABe73 were evaluated in vitro on hepatocyte-like HepaRG cells and in vivo in mice showing low systemic toxicity. Two different fluorescence probes, 1,1′-dioctadecyl-3,3,3′,3′-tetramethylindodicarbocyanine perchlorate (DiD oil) and 1,1′-dioctadecyl-3,3,3′,3′-tetramethylindotricarbocyanine iodide (DiR), were encapsulated to visualize their uptake into HepaRG cell nanoparticles with a strong enhancement of the derived micelles carrying the RGD peptide, with a 10-fold increase compared with PEG42-b-PMLABe73. In vivo in the peritoneal/liver area, a significantly higher fluorescence (double intensity on average) was observed in mice injected with PEG-b-PMLABefor RGD peptide-labeled micelles. Jang et al. synthesized an amphiphilic diblock copolymer, a-carboxyl poly(ethylene glycol)-poly(trimethylene carbonate) (HOOCPEG-PTMC), by ring-opening polymerization [139]. The flexible, high-molecular weight poly(trimethylene carbonate) (PTMC) can represent a valid alternative to ε-caprolactone and l-lactide, not releasing drugs upon its degradation [140]. The cRGD peptide was conjugated to an NHS-activated PEG segment. The nanoparticles prepared had an average diameter of about 50 nm and the same shape and were loaded with PTX. The cRGD specific uptake was detected by comparing the IC_50_ values on U87MG cells, 0.051 mg/mL for MNP/PTX and 0.022 mg/mL for c(RGDyK)-MNP/PTX. In vivo, c(RGDyK)-MNP/PTX tripled the blood circulation time of PTX. Enhanced accumulation of decorated micelles was detected by fluorescence images of tumor tissue in an in vivo xenograft tumor-bearing model. Shia et al. prepared a polymer–PTX conjugate for gastric cancer therapy [141]. The drug was coupled to disulfide-containing linkage dithiodipropionic anhydride (DTDPA) to be released in a reductive tumoral environment, whereas cRGD was linked to the *N*-terminus of PEG. RGD-PEG-SS-PTX micelles (RGD@Micelles) were highly stable in aqueous solution with a CMC value of 2.6 μg·mL^−1^. Decorated peptide FITC-loaded micelles were accumulated in human gastric carcinoma drug-resistant SGC7901/ADR cells, inhibiting cell proliferation and inducing apoptosis with more efficacy compared to non-target micelles. In vivo experiments on BALB/c mice indicated a tumor inhibitory rate of 96.8% for RGD@Micelles compared with 39.4% and 64.7% for the PTX and the undecorated micelles, respectively. Moreover, at 24 h post-injection, the metabolic rate of the RGD@Micelles was lower in the tumor than the two other administrations; the increased retention enhanced the therapeutic efficacy.

Recently, Lu et al. developed a redox-responsive amphiphilic polymer based on a disulfide bond-conjugated prodrug polymer containing CPT and PEG with further modification of internalizing RGD peptide (iRGD) [142]. A tumor-derived protease cleavage exposes the CendR motif that is able to recognize neuropilin-1 (NRP-1) overexpressed on the surface of glioma cells. Thus, the iRGD-functionalized polymeric prodrug micelle system could achieve high tumor penetration and transcytosis for advanced glioma therapy. Meanwhile, the encapsulation of photosensitizer IR780 into micelles effectively combined the chemotherapy of CPT with photodynamic therapy. Additionally, the obtained micelles displayed good stability with controlled drug release behavior, showing great promise for the treatment of brain tumors. The survival rate also confirmed the improved efficacy of glioma treatment by applying CPD@IR780 micelles with laser irradiation. The survival time showed that mice treated with CPD@IR780 micelles with laser irradiation displayed the longest median survival time (49 days), while PBS, CPT, CPC@IR780 and CPD@IR780 micelle treatments achieved the median survival times of 29, 31, 31 and 38 days, respectively.

More recently, with the aim to integrate diagnosis and therapy within a single platform, Qin et al. developed a single amphiphilic molecule containing: (i) the peptide targeting (ii) a poly(Histidine) chain (H9), linked at the *C*-terminus with amino-carboxylpolyethylene glycol (NH_2_-PEG_8_-COOH), (iii) the caspase-responsive DEVD peptide sequence, (iv) the tetraphenylethene (TPE) fluorophore and hydrophobic moiety [143]. Micelles were loaded with DOX and grafted with RGD peptide (TPR@DOX) to address the nanoaggregates to ανβ3 receptors. The aggregation-induced emission (AIE) characteristics of TPE were used to monitor the loading and release of DOX during the delivery process. Furthermore, TPE, in an in vitro assay in murine mammary carcinoma (4T1) cells, indicates apoptosis following the release and re-aggregation of the fluorophore due to caspase-3 activation.

At the same time, a multifunction platform was employed by Bai et al. [144]. They developed a tool with pH- and redox-triggered drug release and aggregation-induced emission (AIE) active imaging. The nanoplatform was designed to achieve an active targeting of SSTR2 receptors overexpressed by neuroendocrine neoplasms (NENs). The octeotide OCT was conjugated to HOOC-PEG-b-PBLG. Then, the hydrophobic moiety was modified by TPE-SS-NH_2_ and further decorated with 3,4-dihydroxyphenethylamine and 3-aminobenzeneboronic acid. Through self-assembly with an average diameter of 274.6 nm and spherical morphology, a multifunctional core-cross-linked hybrid micelle system based on OCT-PEG-b-PGu(DA-TPE) was efficiently obtained and loaded with etoposide (ETO). Etoposide demonstrated a dual-responsive triggered intracellular drug release and an in vitro improved tumor suppression ability. In a mouse xenograft model, free ETO and ETO-loaded micelles were tested. Significant inhibition of the tumor growth (with tumor volumes of 374 and 480 mm^3^) was reported for micelles. The formulation reduced side effects too, as deducted from the histological analysis of the organs.

π–π stacking interactions between aromatic rings on the polymer chain and drug can produce a high micellar stability coupled with an improved drug loading capability. On this statement, Qiao et al. [145] proposed a micelle formulation of poly(b-benzyl malate)-b-polyethylene glycol (PBM-PEG) with a loading capacity of (>20 wt%) of DOX. The systems was formulated to be target-selective, anchoring the Tat peptide to a 2 kDa PEG fragment (PBM-PEG2k-Tat). Mixed micelles were obtained, adding to the PBM-PEG2k-Tat a second amphiphilic copolymer (PBM-pp-PEG5k) containing a long-chain PEG (M.W. 5 kDa) anchored via an MMP-2-sensitive peptide. The Tat moiety was protected under the long-chain PEG in physiological conditions (low MMP-2 concentration 100 ng·mL^−1^), while it was exposed after reaching the tumor district (high MMP-2 concentration 100 ng·mL^−1^). In this manner, the breakage of the peptide enhanced the tumoral cell internalization. In vitro and in vivo results displayed superior toxicity, minor tumor growth and less body loss after treatment with mixed micelles (ratio 1/10) compared to self-assembled micelles of both copolymers and free DOX.

Zhang et al. developed a multifunctional mixed micellar system consisting of glycyrrhetinic acid (GA) for specific liver targeting, Tat peptide for potent cell penetration and pH-sensitive poly(β-amino ester) polymers for acidic-triggered DOX release [146]. These authors also successfully tested DOX/GA@TAT-M internalized by the effect of GA targeting and TAT, protected by the long PEG chain, penetrating HCC cells with enhanced cytotoxicity. DOX release was facilitated (86.5% vs. 45.7% over 144 h) as the result of rapid nanoparticle collapse in the endo/lysosomal environment (pH < 6), with enhanced nuclear delivery of DOX. Additionally, the authors demonstrated that both a prolonged circulation time and enhanced accumulation in the tumor helped its potent tumor growth inhibition activity in vivo. In Table 2, polymeric micelles are listed.

## 5. Conclusions and Perspective

The innovative administration method of active principles is one of the weapons in the fight against cancer. At this time, we surrounded our awareness on micelles achieved by self-assembling or mixing peptide-grafted surfactants or peptide-decorated amphiphilic copolymers. Nanovectors loaded with hydrophobic or hydrophilic cytotoxic drugs or with gene silence sequences and externally functionalized with natural or synthetic peptides were described based on their formulation and in vitro and in vivo behaviors. A crucial challenge is certainly related to precision medicine as it improves the efficacy and reduces the side effects, delivering the drug only to the district where it has to act. In this landscape, micelles can contribute to addressing not only hydrophobic molecules such as PTX but also molecules with a low lifetime in vivo such as SiRNA for genic therapy. Moreover, these systems can be exploited for dual delivery, demonstrating the synergistic effects of small molecules and nucleic acids. Most of the polymeric micelles are sensitive to the cancer environment, releasing the payload under the influence of pH variation or enzyme action. Active targeting by means of drug-encapsulated micelles decorated with targeting bioactive moieties represents the next frontier in drug delivery. In this article, we have reviewed more than 80 systems, most of them formulated in the last decade, decorated with targeting peptides able to recognize overexpressed receptors or with sequences able to favor cell penetration. CPPs in recent formulations have been improved, hindering their function in normal tissue and exploiting their ability only in the cancer tissues. In vivo trials in all reviewed systems demonstrate an increase in mice survival and a reduction in the tumor size. However, it remains equally evident that despite attempts to build multifunctional systems and the promising in vitro and in vivo results, at least until now, peptide micelles have not reached the clinical phases. One issue that hampered the transfer from the laboratory to clinical use is the storage and the reconstruction of nanosystems. The next challenge will be to design micelles that can be more easily formulated and aggregates able to be stored after lyophilization, which can produce similar positive results, significantly increasing the response to the current drugs.

## Figures and Tables

**Table 1:** Peptide surfactant micelles.

Peptide Sequence	Amphiphilic Mol.	Tumor Target	Ref.
CCK8: DYMGWMDF	(C_18_)_2_(ADA)_5_CCK8	CCK2-R-overexpressing cells	[58]
CCK8: DYMGWMDF	(C_18_)_2_(ADA)_5_CCK8	CCK2-R-overexpressing cells	[59]
	(C_18_H_37_)_2_Lys(DTPAGlu)		
CCK8: GDYMGWMDF	(C_18_H_37_)(ADA)_5_-G-CCK8	CCK2-R-overexpressing cells	[60]
	(C_18_H_37_)_2_Lys(DTPAGlu)		
CCK8: DYMGWMDF	[C_18_CysL_5_CCK8]_2_	CCK2-R-overexpressing cells	[61]
OCT: (fCFwLTCT-ol)	OCA-DTPAGlu	SSTR-R-overexpressing cells	[62]
	OCA-DOTA		
OCT: (fCFwLTCT-ol)	(C_18_)_2_(ADA)_5_OCT	SSTR-R-overexpressing cells	[63]
	(C_18_)_2_DTPAGlu/DOTA		
RGD	(C_18_)_2_(ADA)_5_RGD	A2058 melanoma cells	[64]
	(C_16_)_2_(ADA)_5_RGD		
RGD:cRGDfK	(C_18_)_2_(ADA)_5_cRGD	A2058 melanoma cells	[65]
	(C_18_)_2_(ADA)_5_RGD		
RGD:KKGRGDS	(C_12_)KKGRGDS	HepG2-HeLa	[66]
RGD:R7RGDS	C_18_H_5_R_7_RGDS	HepG2	[67]
CCK8: DYMGWMDF	(PDA)(ADA)_2_CCK8	CCK2-R-overexpressing cells	[70]
	(PDA)DTPAGlu		
RGD:KGGGKRGDK	(PDA)(Nph)KGGGKRGDK	SKOV-3 cells	[71]
	(PDA)KGGGKRGDK		

**Table 2:** Polymeric micelles.

Polymer	Peptide	Tumor Target	Drug	Ref.
RGD-PEG-PLA	c(RGDyK)	SLK endothelial cells	SPIO-DOX	[74]
cRGD PEG-PLA	c(RGDyK)	HUVEC	CA4	[75]
PEG-PLA-Dau–cRGD PEG-PLA	c(RGDyK)	C6 glioma cells	DAU	[76]
c(RGDyK)-PEG-PLA	c(RGDyK)	U86MG glioblastoma	PTX	[77]
s-RGD-PEG-PLA	s-RGD	U86MG glioblastoma	PTX	[78]
RGD-PEG-PLA	RGD	B16F10	DOX CA4	[79]
tLyp-1-PEG-PLA	CGNKRTRGC	C6 glioma cells-HUVEC	PTX	[80]
AP-PEG-PLA/MPEG-PAE	AP peptide (CRKRLDRN)	MDA-MB231	DOX	[81]
OCT-PEG-PLA	fCFwLTCT-ol	NCI-H446	Dil-DTX	[82]
NGR-PEG-PLA	NGR	HT1080	DTX	[83]
Tat-PEG-PLA-pHIS/pHIS-PEG	GCGGGTGRKRRQRRR	A2780/AD-MCF7	DOX	[84]
		A549-KB		
pGLU-pPLA/DSPE-PEG-Tat	GCGGGTGRKRRQRRR	A375	DOX	[85]
cRGD-PEG-SS-PCL/PEG-SS-PCL	c(RGDyK)	U86MG glioblastoma	DOX	[86]
		MCF 7		
cRGD PEG-DTC-PCL	c(RGDyK)	U86MG glioblastoma	DOX	[87]
cRGD-PEG-b-PBCL/p160	cRGDfK/VPWXEPAYQRFL	MDA-MB-435	PTX	[88]
cRGD-PEG-b-PCL/p160				
11-PEG-b-PBCL 11–PEG-b-PCL	RGDPAYQGRFL	MDA-MB-435MDA-MB-231, MCF-7	PTX	[89]
18-PEG-b-PBCL 18–PEG-b-PCL	WXEAAYQRFL			
cRGD4C-PEG-b-PCL	KACDCRGDCFCG	MDA-MB-435/LCC6MDR1	SiRNA-DOX	[91]
TAT-PEG-b-PCL	CGRKKRRQRRR			
OCT-PEG-b-PCL	fCFwLTCT-ol	MCF-7	PTX-SAL	[92]
LAN-PEG-b-PCL	(D2NaI)CYWKVCT	H446-MCF-7	PTX	[93]
ALMWP-PEG-PCL	ALMWP	C6 glioma	PTX	[94]
MPEG-PCL-SS-Tat	GRKKRRQRRR	COS7 S-180	pDNA	[95]
MPEG-PCL-SS-Tat	GRKKRRQRRRCG	S-180	siVEGF	[96]
MPEG-PCL–CH_2_R_4_H_2_C	CH_2_R_4_H_2_C	S-180	siVEGF	[97]
MPEG-PCL-CH_2_R_4_H_2_C	CH_2_R_4_H_2_C	B16F10	siRelA	[98]
MPEG-PCL-Tat	GRKKRRQRRRG	C6 glioma	CUM	[99]
MPEG-PCL-Tat	GRKKRRQRRRG	C6 glioma	siRNA-CPT	[100]
MPEG-PCL-Tat	GRKKRRQRRRG	RN33B	siRNA	[101]
MPEG-PCL-Tat; Steroyl-Bom	GRKKRRQRRRPPQ	C6 glioma	CPT CUM	[102]
	GQWAVGHLM			
RGD4C-PEG-b-P(CL-g-DP)	KACDCRGDCFCG	MDA435/LCC6	DOX-SiRNA	[106]
TAT-PEG-bP(CL-g-DP)	CGRKKRRQRRR			
cRGD-PEG6k-P(DTC-co-CL), TAT-PEG2k-P(DTC-co-CL)	C(RGDfK)	U87MG glioma cells	DTX	[107]
	YGRKKRRQRRRC			
DCL-PEG-b-PCL	YGRKKRRQRRR	PCa cells	SiRNA-DTX	[108]
TAT-PEG-b-P(CL-g-SP)				
cRGD-PEG-b-(PLL-IM)	c(RGDyK)	HeLa-luc	SiRNA	[111]
cRGD-PEG-PLL(MPA)	c(RGDyK)	HeLa-Luc	SiRNA	[112]
RGD-PEG-p(Lys)-p(Leu)	RGD	MDA-MB-231-MCF7	DCPT	[113]
Tat-p(Lys)-p(Leu)	YGRKKRRQRRR	HELA	DOX	[114]
c-RGD-PEG–P(Glu)	c(RGDyK)-RAD	U87MG glioblastoma	DACHPt	[116]
cRGD-PEG–P(Glu)	c(RGDyK)	LNM	DACHPt	[117]
cRGD-PEG–P(Glu)	c(RGDyK)	HNSCC-CSC	CDDP	[118]
PLG-g-Ve/PEG	cRGD	B16F10	DTX-CDDP	[119]
cRGD-PEG-PLA PEG-PGA)-API	c(RGDyK)	B16F10	DOX	[120]
cRGD-PEG-PDEARGDPEGPAsp	c(RGDfC)	C6	Cd-Te (QD)	[121]
cRGD PEG-b-PBLA	c(RGDfC)	U87MG	Epi	[122]
PEG-GPLGVRGDG-P(BLA-co-Asp)	GPLGVRGDG	HT1080	DOX	[123]
cRGD-PEG-b-PAPA	c(RGDyK)	B16F10	DTX	[124]
cRGD-Lipep-Ms	c(RGDyK)	HCT-116	MMAE	[126]
c(RGD)-PF127	c(RGDyK)	MDR KBv	DOX-PTX	[127]
c(RGD)-PF127	c(RGDyK)	U87MG	DOX-PTX	[128]
Pf127-D4-(AdA)_2_(C18)_2_	LARLLT	HepG2	-	[130]
cRGDfK	cRGDfK	B16 t	Quercitin	[131]
cRGDfK	cRGDfK	B16F10	Anlotinib	[132]
CCK8-DSPE-PEG_2000_	DYMGWMDF	-	Gd	[133]
(C18)_2_DTPAGlu-Gd				
LAN-DPSE-PEG_2000_	(D2NaI)CYWKVCT	H446	PTX	[134]
GE11-DSPE-PEG_2000_	YHWYGYTPQNVI	Hep-2	PTX	[135]
GE11-L-DSPE-PEG_2000_	YHWYGYTPQNVI	SMMC-7721 c	DOX	[136]
RGDBiot-StreptBiot-PEG62-b-PMLABe73	c(RGDyK)	HepaRG	DiD DiR	[138]
cRGD-PEG-PTMC	c(RGDyK)	U87MG	PTX	[140]
c-RGD-PEG-SS-PTX	c(RGDyK)	SGC7901	PTX	[141]
iRGD-PEG-SS-CPT	CRGDRGPDC	U87MG	CPT-IR780	[142]
RGD-PEG-H_9_-DEVD-TPE	RGD	4T1	DOX-TPE	[143]
OCT-PEG-b-PGu(DA-TPE)	fCFwLTCT-ol	LCC-18-NCH-446 c	ETO	[144]
Tat-PEG-PBM	YGRKKRRQRRR	HT1080, MCF-7 MDA-MB-231	DOX	[145]
Tat-PEG1k-PHHD-GA-P5kCV	AYGRKKRRQRRR	SMCC 7721	DOX	[146]

## Data Availability

Not applicable.

## Sample Availability

Samples of the compounds are not available from the authors.
